# Mitochondrial STAT3 exacerbates LPS-induced sepsis by driving CPT1a-mediated fatty acid oxidation

**DOI:** 10.7150/thno.63751

**Published:** 2022-01-01

**Authors:** Rongqing Li, Xueqin Li, Jie Zhao, Fandong Meng, Chen Yao, Ensi Bao, Na Sun, Xin Chen, Wanpeng Cheng, Hui Hua, Xiangyang Li, Bo Wang, Hui Wang, Xiucheng Pan, Hongjuan You, Jing Yang, Takayuki Ikezoe

**Affiliations:** 1 Jiangsu Province Key Laboratory of Immunity and Metabolism, Xuzhou Medical University, Xuzhou, Jiangsu, China.; 2 Department of Pathogenic Biology and Immunology, Xuzhou Medical University, Xuzhou, Jiangsu, China.; 3 Department of Endocrinology, The First Affiliated Hospital of Xuzhou Medical University, Xuzhou Medical University, Xuzhou, Jiangsu, China.; 4 Department of Critical Care Medicine, The First Affiliated Hospital of Xuzhou Medical University, Xuzhou Medical University, Xuzhou, Jiangsu, China.; 5 Department of Infectious Disease, The First Affiliated Hospital of Xuzhou Medical University, Xuzhou Medical University, Xuzhou, Jiangsu, China.; 6 Department of Hematology, Fukushima Medical University, Fukushima, Japan.

**Keywords:** mitochondrial STAT3, FAO, CPT1a stabilization, USP50

## Abstract

**Rationale:** We found that a subset of signal transducer and activator of transcription 3 (STAT3) translocated into mitochondria in phagocytes, including macrophages isolated from individuals with sepsis. However, the role of mitochondrial STAT3 in macrophages remains unclear.

**Method:** To investigate the function of mitochondrial STAT3 *in vivo*, we generated inducible mitochondrial STAT3 knock-in mice. A cytokine array analysis, a CBA analysis, flow cytometry, immunofluorescence staining and quantification and metabolic analyses *in vivo* were subsequently performed in an LPS-induced sepsis model. Single-cell RNA sequencing, a microarray analysis, metabolic assays, mass spectrometry and ChIP assays were utilized to gain insight into the mechanisms of mitochondrial STAT3 in metabolic reprogramming in LPS-induced sepsis.

**Results:** We found that mitochondrial STAT3 induced NF-κB nuclear localization and exacerbated LPS-induced sepsis in parallel with a metabolic switch from mainly using glucose to an increased reliance on fatty acid oxidation (FAO). Moreover, mitochondrial STAT3 abrogated carnitine palmitoyl transferase 1a (CPT1a) ubiquitination and degradation in LPS-treated macrophages. Meanwhile, an interaction between CPT1a and ubiquitin-specific peptidase 50 (USP50) was observed. In contrast, knocking down USP50 decreased CPT1a expression and FAO mediated by mitochondrial STAT3. The ChIP assays revealed that NF-κB bound the USP50 promoter. Curcumin alleviated LPS-mediated sepsis by suppressing the activities of mitochondrial STAT3 and NF-κB.

**Conclusion:** Our findings reveal that mitochondrial STAT3 could trigger FAO by inducing CPT1a stabilization mediated by USP50 in macrophages, at least partially.

## Introduction

Sepsis is a life-threatening condition with limited therapeutic options and is characterized by excessive systemic inflammation and multiple organ failure 1. Macrophages, the principal phagocytic components of the immune system, are among the primary regulators of innate immunity responsible for a broad range of inflammatory processes and play important roles in sepsis pathogenesis 2.

Accumulating evidence has demonstrated that in addition to providing energy and substrates for growth and survival, metabolic reprogramming also plays a critical role in regulating the functions and polarization of macrophages 3-6. In contrast to interleukin-4 (IL-4)-induced alternatively activated macrophages in which an increase in FAO was observed 4, 5, in the presence of LPS, macrophages exhibit impaired mitochondrial oxidative phosphorylation (OXPHOS) and enhanced glycolytic metabolism 6. Mitochondria are key players in the metabolic switch 7. A decrease in the mitochondrial mass is observed in LPS/IFN-γ-stimulated macrophages 8. Mitochondrial adaptations contribute to macrophage activation 2, 9, highlighting the importance of the mitochondrial changes in infected macrophages that drive modifications of host metabolism and immunological reprogramming 7.

STAT3 has been implicated in cell proliferation and human cancers 10. In addition to its well-established roles in the nucleus, a new noncanonical role of STAT3 has been discovered in mitochondria. Mitochondrial STAT3, a regulator of Ca^2+^ homeostasis, affects the mitochondrial production of ROS 11. Moreover, mitochondrial STAT3 increases OXPHOS independent of its nuclear activity 12. Mitochondrial STAT3 also elevates the mitochondrial membrane potential and ATP synthesis 13. These observations imply that mitochondrial STAT3 could play a role in mediating mitochondrial metabolism. Therefore, in this study, we utilized inducible mitochondrial STAT3 knock-in mice and discovered that following treatment with LPS, mitochondrial STAT3 exacerbated sepsis along with an increase in FAO via the stabilization of CPT1a.

## Methods

### Mice

C57BL/6 (B6) (CD45.2) and *Ubc^ERT2Cre/+^* mice were obtained from Shanghai Model Organisms Center, Inc. (Shanghai, China). The *Lyz^Cre/+^* mice were a kind gift from Professor Hui Wang (Xuzhou Medical University, China). The *Rosa26^LSL-MLS-STAT3^* knock-in mice were generated at the Shanghai Model Organisms Center, Inc. Briefly, the COX IV sequence of yeast was utilized as the mitochondrial localization sequence (*MLS*). The CAG-LSL-*MLS*-*Stat3*-WPRE-pA vector was inserted into the Gt(ROSA)26Sor (*Rosa26*) locus by the CRISPR-Cas9 technique. To eliminate off-target effects, the knock-in mice were backcrossed onto a C57BL/6 background for three generations. All mouse strains were genotyped by PCR using the following primers: wild-type allele (994 bp), P1: 5'-TCAGATTCTTTTATAGGGGACACA-3', P2: 5'-TAAAGGCCACTCAATGCTCACTAA-3', knock-in allele (414 bp), P3: 5'-GCGTATCCACATAGCGTAAAAGG-3', P4: 5'-TACCCCGACATTCCCAAGG-3'. To induce mitochondrial STAT3 expression, adult mice were given 75 mg/kg 4-hydroxytamoxifen (4-OHT) solution every other day 7 times by i.p. injections. The mice were strictly bred and maintained under protocols approved by the Institutional Animal Care and Use Committee of Xuzhou Medical University (Approval No. 202104A075).

### Sepsis model

The mice were intraperitoneally injected (i.p.) with phosphate-buffered saline (PBS) or LPS (20 mg/kg, Sigma). For the curcumin treatment experiment, the mice were injected (i.p.) with 10 mg/kg curcumin (MCE, Shanghai, China) 1 h prior to the LPS injection.

### Human samples

Approximately 3 mL whole blood were collected in K2-EDTA blood collection tubes (Rich Science, Chengdu, China) from two healthy male volunteers and patients diagnosed with sepsis who provided informed consent according to the protocol approved by the Scientific and Ethics Committee (IMSS reference number XYFY2021-KL248-01). Peripheral blood mononuclear cells (PBMNCs) were isolated from peripheral blood by Ficoll gradient centrifugation (Amersham Biosciences, Freiburg, Germany). Then, the PBMNCs were suspended in RPMI 1640 cell culture media and incubated for 2 h in 5% (v/v) CO_2_ at 37 °C to allow the attachment of mononuclear phagocytes to the bottom plastic surface as previously described 14, 15. The nonadherent cells were removed. The mononuclear phagocytes were washed with prewarm PBS (Kaiji, Nanjing, China) and collected for the indicated experiments. After some of the sample was fixed in 10% neutral buffered formalin for immunofluorescence, the remaining sample was subjected to mitochondrial protein extraction.

### Cell isolation and culture

Primary mouse embryonic fibroblasts (MEFs) were generated and cultured as previously described 16.

BM-derived macrophages were generated as previously described 17, 18. Briefly, lineage-committed cells were depleted using FITC-anti-CD3 (IC: FITC-Rat IgG2b, κ, 17A2, BioLegend), FITC-anti-CD19 (IC: FITC-Rat IgG2a, κ, 6D5, BioLegend), FITC-anti-Ly6G (FITC-Rat IgG2a, κ, 1A8, BioLegend) and FITC-anti-TER119 (, IC: FITC-IgG2b, κ, TER-119, eBioscicence) antibodies. Lineage^-^ whole BM cells were cultured in DMEM supplemented with 10% FBS and 20 ng/mL murine recombinant macrophage-colony stimulating factor (M-CSF, Peprotech) for 4 days.

### Preparation of the subcellular fractions

The nuclear, cytoplasmic, and mitochondrial fractions were prepared by using a mitochondria isolation kit (Beyotime, Shanghai, China). Briefly, MEFs were lysed. The homogenate was centrifuged at 600 g at 4 °C for 10 min to obtain a supernatant containing mitochondria and cytosolic fractions, followed by centrifugation at 11,000 g at 4 °C for 10 min to collect mitochondrial pellets. The supernatant fraction was the cytosolic protein fraction. After washing, RAPI was added to the pellets, and the supernatant (nuclear fraction) was collected.

### Flow Cytometry

For the peripheral blood analysis, red blood cells (RBCs) were lysed with RBC lysis buffer (FCMACS, Nanjing, China). Then, the cells were stained with Percp-Cy5.5-anti-CD45 (IC: PerCP-Cy5.5-IgG2b, κ, 30-F11, BD Biosciences, CA, USA), FITC-anti-CD3, V450-anti-CD19 (IC: V450-IgG2a, κ, 1D3, BD Biosciences), BV421-anti-Ly6G (IC: BV421-Rat IgG2a, κ, 1A8, BioLegend), and PE-anti-F4/80 (IC: PE-Wistar (outbred) IgG2a, κ, T45-2342, BD Biosciences) and incubated at 4 °C for 15 min.

For the BM progenitor analysis, after lysing the RBCs, nonspecific antibody binding to the cells was blocked by incubation with an anti-CD16/32 antibody (2.4G2, BD Biosciences), and then, the samples were stained with other antibodies. The lineage markers used in the BM progenitor analysis included CD3, CD19, Ly6G, Ter-119, B220, CD11c, and CD11b as previously described 19. The cells were analyzed using a FACSAria flow cytometer (BD Biosciences). The data were analyzed using FlowJo (FlowJo LLC).

For the macrophage analysis in lung tissues, at the end of the experiments, lung tissues were removed, and single-cell suspensions were prepared as previously described 20. Briefly, the lung tissues were digested with RPMI-1640 culture medium containing 1 mg/mL collagenase IV (VICMED), 20 ng/mL DNase I (VICMED), and incubated at 37 °C for 45 min with gentle vortexing every 8-10 min. After washing, the cells were strained through a 70 μm cell strainer and lysed with RBC lysis buffer. Subsequently, the cells were stained with 7-AAD (559925, BD) to label the dead cells, Percp-Cy5.5-anti-CD45, BV421-anti-Ly6G, FITC-anti-CD11b, PE/Cy7-anti-CD11c (IC: PE/Cy7-Armenian Hamster IgG1, λ2, HL3, BD bioscience), PE-anti-F4/80, and APC-anti-CD64 (APC-mouse IgG1, κ, X54-5/7.1, Elabscience) for 15 min and subjected to FACS for the analysis.

### Cell sorting

For the BM progenitor cell sorting, BM cells were stained with PE/Cy7-anti-CD16/32 (IC: PE/Cy7-Rat IgG2a, λ, 93, BioLegend) and incubated at 4 °C for 15 min before staining with PE/Cy5-anti-c-Kit (IC: PE/Cy5-Rat IgG2b, κ, 2B8, BioLegend), APC/Cy7-anti-Sca1 (IC: APC/Cy7-Rat IgG2a, κ, D7, BioLegend), , and PE-anti-CD34 (IC: PE-Rat IgG2a, κ, RAM34, BD Biosciences) at 4 °C for 25 min. FACS was performed using a BD FACS Aria III (BD Biosciences) to achieve > 95% purity.

### Single-cell RNA-sequencing (scRNA-seq)

c-Kit^+^ cells from BM were isolated by FACS. A single-cell RNA-seq experiment was performed by experimental personnel in the laboratory of GENECHEM. (Shanghai, China) as previously described 21, 22. A table containing the molecule counts per gene per cell was the output. The gene expression profiles of 4,974 and 5,117 cells were recovered from the BM of *Rosa26^LSL-MLS-Stat3^* or *Rosa26^LSL-MLS-Stat3^*;*Ubc^ERT2Cre/+^* mice, respectively, with 46,100 and 42,302 mapped reads per cell. The analysis of the single-cell transcriptome profiles was performed using BD Data View.

### Microarray analysis

BMDMs were treated with 100 nM 4-OHT for 3 days, followed by incubation with either control diluent or 100 ng/mL LPS for 6 h. The total RNA was isolated from the sorted cells using TRIzol reagent (Invitrogen) according to the manufacturer's protocol. The library preparation, clustering and sequencing were performed by Majorbio (Shanghai, China).

### Metabolic analyses *in vivo*

The *Rosa26^LSL-MLS-Stat3^* and *Rosa26^LSL-MLS-Stat3^*;* Ubc^ERT2Cre/+^* mice were treated with 4-OHT 7 times, followed by LPS injection. The mice were placed individually in metabolic chambers of an Oxymax system (Columbus Instruments) and allowed to acclimate for 24 h before (Day 0) and after (Day 1) the LPS injection.

### Metabolic assays

BMDMs** (**8.0 × 10^4^) were seeded into 24-well Seahorse microplates in 250 μL of growth medium and incubated at 37 °C at 5% CO_2_ for 16 h, followed by treatment with control diluent or 100 ng/mL LPS for 6 h. Before starting the test, the cells were washed twice with assay running media and equilibrated in a non-CO_2_ incubator. The extracellular acidification rate (ECAR) and the oxygen consumption rate (OCR) were measured as previously described 23. To measure exogenous and endogenous FAO, BMDMs were cultured in substrate-limited DMEM containing 0.5 mM glucose, 1 mM GlutaMAX, 0.5 mM carnitine, and 1% FBS overnight. On the following day, the medium was replaced with FAO assay medium, and the oxygen consumption rate (OCR) was measured in real time using a Seahorse XF24 Extracellular Flux Analyzer (Seahorse Bioscience). All results were analyzed using Wave software version 2.4.0 (Agilent).

### Histological analysis

Tissues were fixed overnight in 10% formalin (buffered to neutral). The formalin-fixed tissues were paraffin-embedded at the Histopathology Core Facility. Sections (5 mm) were cut and stained with hematoxylin and eosin (H&E).

### Immunofluorescence staining and Confocal Microscopy

Immunofluorescence staining was performed with anti-CD68 (25747-1-AP, Proteintech, 1:200) using a PANO 4-plex IHC kit (Yuanxi, Shanghai, China) as previously described 19. MEFs were stained with anti-p-STAT3 (Tyr705) (D3A7, cat# 9145, 1:200), anti-p-STAT3 (Tyr727) (cat# 9134, 1:200), anti-HSP60 (D6F1, cat# 12165, 1:1400) using the APExBIO kit (APExBIO, Houston, USA). Anti-p-STAT3 (Tyr705), anti-p-STAT3 (Tyr727) and anti-HSP60 antibodies were purchased from Cell Signaling Technology (MA, USA). The analyses were carried out under a Zeiss LSM880/800 confocal microscope.

### Mass spectrometry

Protein samples were incubated with anti-flag M2 magnetic beads (M8823, Sigma). After washing, the samples were separated on 4-12% gradient sodium dodecyl sulfate polyacrylamide gel electrophoresis (SDS-PAGE) gels and stained using Coomassie blue stain as previously described 24. The gel lanes were cut and harvested. The gel digestion and peptide analysis were performed by PuboBio (Xuzhou, China). Briefly, the gels were digested in solution by adding 1% trypsin and incubated at 37 °C overnight. The obtained peptides were analyzed by a nanoLC.2D (Eksigent Technologies) coupled with a TripleTOF 5600+ System (AB SCIEX, Concord, ON) as previously described 25. The original MS/MS data were submitted to ProteinPilot Software (version 4.5, SCIEX) and searched against the UniProt Human database (04/08/2019, 20,419/559,228 entries searched). The search results were exported from ProteinPilot for summarization, validation, and comparison as previously described 25.

### Immunoblot assay

To block CPT1a degradation, cells were treated with or without the proteasome inhibitor MG-132 (final concentration: 5 μM; Selleck, Houston, Texas, USA) for 8 h. The procedures and conditions of the IP and immunoblot assays were performed as previously described 16. Briefly, cell lysates were extracted and separated on an 8% SDS-PAGE gel. After semidry transfer, the membranes were sequentially probed with the indicated antibodies. For IP, lysates were prepared in IP lysis buffer (Thermo) containing a protease inhibitor cocktail (Abm) and incubated with an anti-CPT1a (cat# 15184-1-AP, 3.5 μg/mg) antibody with gentle shaking at 4 °C overnight. On the following day, 20 µL of protein A/G-agarose (Thermo Fisher Scientific) were added to each IP sample, and the samples were incubated for another 1 h. Subsequently, the beads were washed, resuspended in 30 µL of 1× loading buffer, boiled for 3 min, and subjected to Western blot detection. Anti-p-STAT3 (Tyr705) (1:2000), anti-p-STAT3 (Tyr727) (1:2000), anti-STAT3 (D3Z2G, cat# 12640, 1:2000), anti-histone H3 (cat# 9715, 1:3000), anti-HSP60 (1:2000) and anti-p65 (D14E12, cat# 8242, 1:2000) were purchased from Cell Signaling Technology (MA, USA). Anti-USP50 (cat# 20374-1-AP, 1:2000), anti-CPT1a (1:2000), anti-CPT2 (cat# 26555-1-AP, 1:2000), anti-Ub (cat# 10201-2-AP, 1:2000), anti-HA (51064-2-AP, 1:2000) and anti-β-actin (cat# 66009-1-Ig, 1:3000) were obtained from Proteintech. Anti-α-tubulin (cat# 66031-1-Igsc-5286, 1:200) was purchased from Santa Cruz (Texas, USA). Anti-p-p65 (Ser536, AF2006, 1:500, Changzhou, China) was obtained from Affinity (Wuhan, China). Anti-Myc (cat# 02060, 1:1000) was purchased from Abbkine (Wuhan, China). Anti-flag (cat#, 1:1000) was purchased from Abmart (Shanghai, China). The bands of indicated protein were quantified after normalization to actin and compared using ImageJ software.

### siRNAs, Plasmid DNA and transfection

siRNAs against USP50 were purchased from Jima (Shanghai, China). Flag-tagged human CPT1a and flagged-tagged human USP50 plasmid DNAs were purchased from GeneChem (GeneChem, Shanghai, China). HA-Ubquitin was obtained from Professor Hongjuan You. Human USP50 cDNA was amplified and cloned into the pcDNA3-3myc vector. HEK293T cells were transfected with the indicated plasmid DNA utilizing jetPRIME transfection reagent (Polyplus, France) according to the manufacturer's instructions. After 24 h, the cells were harvested and subjected to the indicated experiments.

### *In vivo* ubiquitination assay

The* in vivo* ubiquitination assay was performed as previously described 26. A Western blot analysis was performed to probe the ubiquitinated forms of CPT1a.

### Protein half-life assay

The procedures and conditions of the CPT1a protein half-life assay were performed as previously described 26. The CPT1a bands were quantified after normalization to actin and were plotted as the relative amount of protein remaining compared with the 0 min treatment time. The bands were compared quantitatively using ImageJ software.

### Real-time RT-PCR

The total RNA was isolated and subjected to real-time RT-PCR using SYBR Green I Master Mix (Roche Diagnostics Gmbh) on a LightCycler 480 system (Roche Group) as previously described 19. The primers used for real-time RT-PCR are shown in Table 1.

The mtDNA quantification was performed as previously described 27. The primer sequences are provided in Table 1.

### ChIP assay

The binding sites of NF-κB on the USP50 promoter were predicted, and a ChIP analysis was performed using a commercially available kit (Beyotime Biotechnology) as previously described 19. The primer sets used for the amplification of the USP50 promoter region between -916 and -780 bp were as follows: forward 5'-CTCTCTGCCTGCTCTCTGCT-3', reverse 5'- ACTAAAAGCATGGGGCTGTG-3'. The samples were separated by electrophoresis on a 2% agarose gel and visualized by ethidium bromide staining.

### Cytokine array analysis

Serum harvested from either Rosa26^LSL-MLS-Stat3^ or Rosa26^LSL-MLS-Stat3^; *Ubc^ERT2Cre/+^* mice was analyzed using a RayBio® Mouse Cytokine Antibody Array 3 (RayBiotech, AAM-CYT-G3-8).

### Cytometric bead array (CBA)

The cytokine concentrations were measured by a cytometric bead array (CBA) by using a CBA kit (BioLegend) according to the manufacturer's protocol.

### ATP, Lactate dehydrogenase (LDH) and Albumin analysis

The levels of ATP were examined using enhanced ATP assay kit (Beyotime, Shanghai, China). The levels of LDH and albumin were measured with an LDH assay kit (Nanjing Jiancheng Bioengineering Institute, Nanjing, China) and an albumin detection kit (Nanjing Jiancheng Bioengineering Institute) according to the manufacturer's protocol, respectively.

### Enzyme-linked immunosorbent assay (ELISA)

The concentrations of MCP-5 in the serum were examined using ELISA kits (Jiangsu Meimian industrial Co., Ltd, Yancheng, China) according to the manufacturer's instructions.

### Statistical analysis

The statistical analysis assessing differences was performed using an unpaired Student's t-test, Multiple t test- one per row or two-way ANOVA followed by multiple comparisons by Prism statistical analysis software (GraphPad Software, San Diego, CA). The data are presented as the mean ± SD. Significance is indicated as follows: ** *P* < 0.01, * *P* < 0.05 or n.s. for not significant.

## Results

### STAT3 localized in mitochondria of infected phagocytes* in vivo*

**Figure 1:**
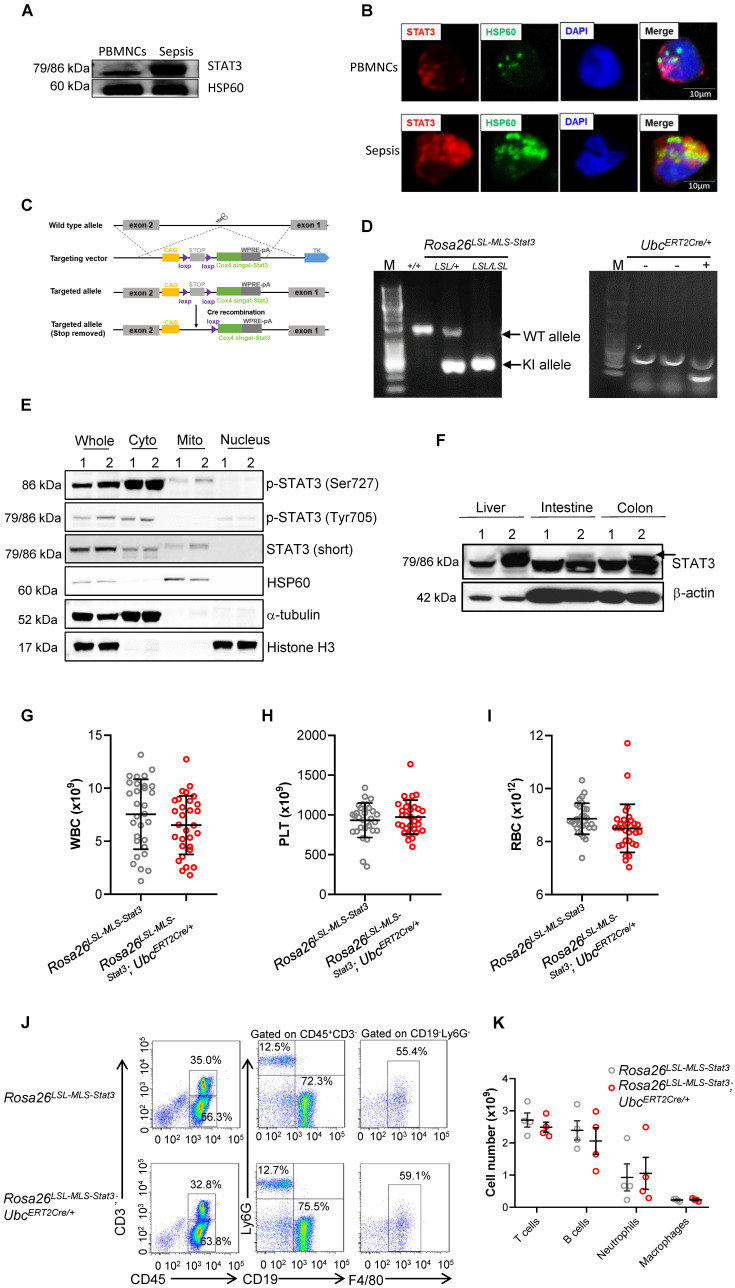
** Generation of inducible mitochondrial STAT3 knock-in mice. (A)** The mitochondria of PBMNCs were extracted from healthy volunteers and sepsis patients. The levels of STAT3 were analyzed by a Western blot analysis. The data shown are from one of three independent experiments. **(B)** Immunofluorescence of STAT3 (red), HSP60 (green), and DAPI (blue) in PBMNCs from healthy volunteers and sepsis patients. The data are representative of one of two independent experiments. **(C)** Schematic representation of the mitochondrial STAT3 gene-targeting vector. The gray boxes represent the first two exons of the *Stat3* gene, and the exon numbers are indicated. The light green box is mitochondrial STAT3. The purple, yellow, and dark gray boxes are selection markers. The triangles indicate *loxP* sites with the orientations noted. **(D)** PCR amplification of genomic DNA isolated from toe snips of mouse pups. **(E)** Early-passage MEFs (passage 2) of *Rosa26^LSL-MLS-Stat3^* and *Rosa26^LSL-MLS-Stat3^;Ubc^ERT2Cre/+^* mice were prepared from embryos (E13.5) obtained by breeding *Ubc^ERT2Cre/+^* mice on the *Rosa26^LSL-MLS-Stat3^* background. Cells were treated with 100 nM 4-hydroxytamoxifen (4-OHT) for 3 days. Nuclear, cytoplasmic, and mitochondrial fractions were extracted. The levels of the indicated proteins were analyzed by a Western blot analysis. 1, *Rosa26^LSL-MLS-Stat3^*; 2, *Rosa26^LSL-MLS-Stat3^;Ubc^ERT2Cre/+^*.** (F)** Six-week-old mice were treated with 4-OHT 7 times. Two days later, the liver, intestine and colon tissues were removed. Protein was extracted and subjected to a Western blot analysis to examine the levels of STAT3 and β-actin. 1, *Rosa26^LSL-MLS-Stat3^*; 2, *Rosa26^LSL-MLS-Stat3^;Ubc^ERT2Cre/+^*. (E and F) The data shown represent one of three independent experiments. **(G-I)** Six-week-old mice were treated with 4-OHT 7 times. Two days later, the total number of WBCs **(G)**, PLTs **(H)**, and RBCs **(I)** in the peripheral blood of either the *Rosa26^LSL-MLS-Stat3^* mice or the *Rosa26^LSL-MLS-Stat3^;Ubc^ERT2Cre/+^* mice was examined by a Sysmex XP-100 hematologic analyzer. The dot graph is represented as the mean ± SD. The data shown (n = 30 mice/group) represent a combination of two independent experiments. **(J)** Six-week-old mice were treated with 4-OHT 7 times. Two days later, the distribution of T cells, B cells, neutrophils, and macrophages in the peripheral blood of mice with different genotypes was assessed by flow cytometry. The data shown are representative of one of two independent experiments. **(K)** The graph shows the number of T cells, B cells, neutrophils, and macrophages in the peripheral blood gating strategy in (J). The data shown are representative of one of two independent experiments (n = 4 mice/group) with similar results. (G-J) Significance was calculated using an unpaired Student's t-test.

We and other researchers have found that upon an LPS challenge, a fraction of STAT3 translocated into the mitochondria of murine macrophages. To determine whether STAT3 localizes in mitochondria in humans, we extracted the mitochondrial fraction of phagocytes from sepsis patients and healthy volunteers and performed SDS-PAGE. After gel digestion, the peptides were analyzed by mass spectrometry, and the original MS/MS data were submitted to ProteinPilot Software (version 4.5, SCIEX) for protein identification. We detected STAT3 peptides in the mitochondrial fraction of phagocytes from sepsis patients and compared them with those from healthy volunteers (**Table 2**). The levels of heat shock protein 60 (HSP60), which was used as a mitochondrial-protein loading control 28, are significantly increased in sepsis patients 29, 30. To compare the levels of mitochondrial STAT3 between the sepsis patients and healthy volunteers, we loaded the same amount of mitochondrial protein for SDS-PAGE. Compared to the healthy volunteers, STAT3 indeed localized in the mitochondria of phagocytes isolated from the sepsis patients, and the mitochondrial STAT3 levels were obviously enhanced in the phagocytes from the sepsis patients (**Figures 1A-B, Figure S1A**).

### Generation of inducible mitochondrial STAT3 knock-in mice

To explore the role of mitochondrial STAT3* in vivo*, we generated inducible mitochondrial STAT3 knock-in mice (**Figure 1C**). After crossing *Rosa26^ LSL-MLS-mSTAT3^* mice with *Ubc^ERT2Cre/+^* mice (**Figure 1D**), STAT3 expression in mitochondria was induced by 4-OHT. MEFs are a type of fibroblasts derived from mouse embryos without cytokine induction. To avoid the effects of cytokines in cultured immune cells on STAT3 phosphorylation and other “off-target” effects, we examined the levels of mitochondrial STAT3 utilizing MEFs. Consistent with our estimation, upon the 4-OHT treatment, the relative phosphorylated forms of STAT3 (Ser727) and the relative amount of STAT3 localized in mitochondria, but not in the cytoplasmic and nuclear fractions, in the MEF cell lysates from the *Rosa26^ LSL-MLS-mSTAT3^*;*Ubc^ERT2Cre/+^* knock-in mice were higher than that in the MEF cell lysates from the *Rosa26^ LSL-MLS-mSTAT3^* mice (**Figure 1E,** Lanes 1 and 2, 5 and 6**, Figure S1B**). The relative amounts of p-STAT3 (Tyr705) were almost identical in those MEF cells (**Figure 1E,** Lanes 1 and 2**,** 3 and 4, **Figure S1B**). To verify the cell fractionation data, we performed immunofluorescence staining and found that p-STAT3 (Ser727) colocalized with HSP60 in 4-OHT treated *Rosa26^ LSL-MLS-mSTAT3^*;*Ubc^ERT2Cre/+^* MEFs (**Figure S1C**). Compared to the *Rosa26^ LSL-MLS-mSTAT3^* MEFs, the levels of p-STAT3 (Ser727) were higher in 4-OHT treated *Rosa26^ LSL-MLS-mSTAT3^*;*Ubc^ERT2Cre/+^* MEFs (**Figure S1C**). In contrast to p-STAT3 (Ser727), MEFs expressing p-STAT3 (Tyr705) were barely to be detected by immunofluorescence staining (**Figure S1C**).

To substantiate the observations in MEFs, we next examined the STAT3 protein levels in mouse tissues. In the *Rosa26^LSL-MLS-mSTAT3^*;*Ubc^ERT2Cre/+^* knock-in mouse tissues, the STAT3 protein levels were increased after the 4-OHT injection (**Figure 1F, Figure S1D**). The elevated STAT3 expression in the tissues harvested from the homogeneous and heterogeneous knock-in mice was almost identical (**Figure S1E**). Therefore, we assumed that it was reasonable to utilize both homogeneous and heterogeneous *Rosa26^LSL-MLS-mSTAT3^*; *Ubc^ERT2Cre/+^* knock-in mice for this study.

The deletion of *Stat3* leads to a decrease in the percentage of long-term marrow repopulating hematopoietic stem cells (LT-HSCs) along with dysregulated mitochondrial function, contributing to a shortened lifespan and a blood phenotype with similarities to the human diseases myelodysplastic syndrome and myeloproliferative neoplasms 10, implying that mitochondrial STAT3 could impair HSC development. However, the findings observed in a previous *Stat3^-/-^* mouse model could be the result of the loss of *Stat3*'s role in mitochondrial function and nuclear transcription factor function. Thus, to exclude the possibility that mitochondrial STAT3 affects HSC development under physiological conditions, we carried out blood cell counts and noticed that the total numbers of white blood cells (WBCs), platelets, and RBCs were not influenced (**Figures 1G-I**). In addition, the percentages and cell numbers of T cells, B cells, neutrophils, and macrophages did not significantly differ (**Figures 1J-K**). Additionally, the percentages and cell numbers of progenitors, including LT-HSCs and GMP, were almost identical (**Figures S2A-C**). These observations indicate that under physiological conditions, mitochondrial STAT3 might not influence HSC development.

### Mitochondrial STAT3 exacerbated LPS-induced sepsis

**Figure 2:**
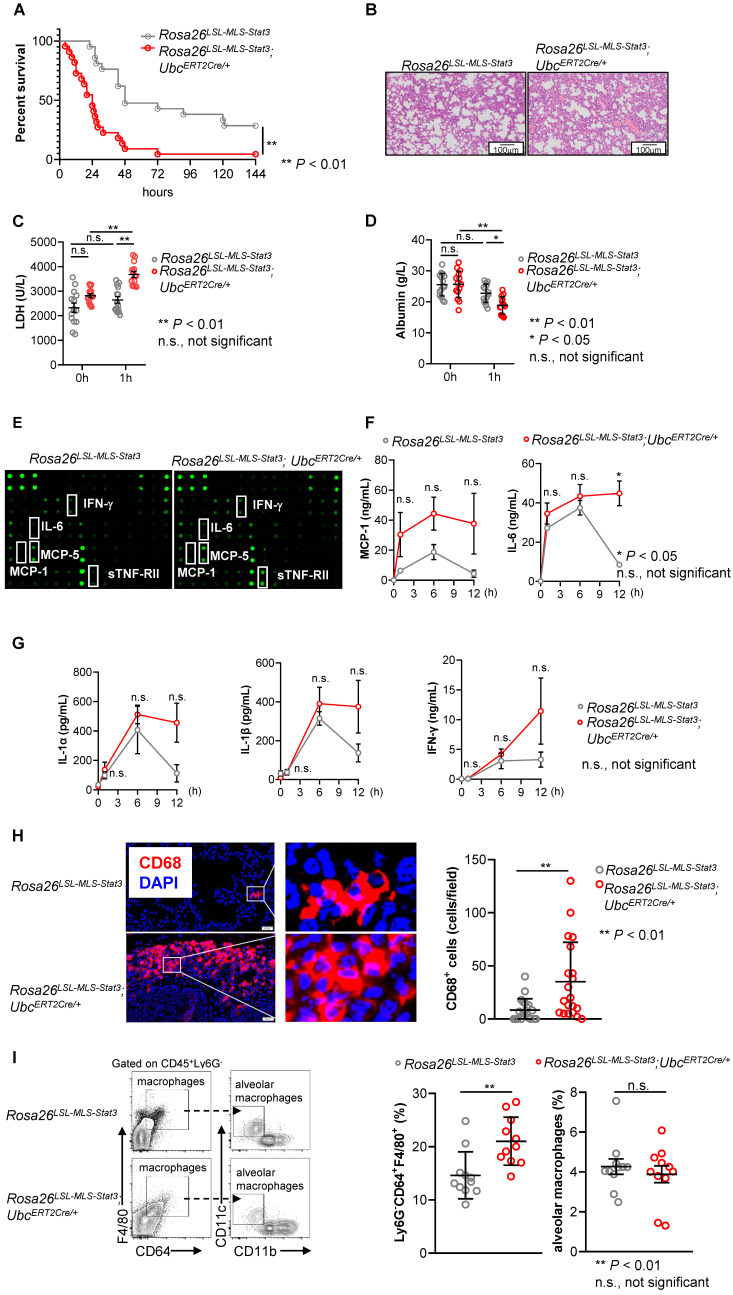
** Mitochondrial STAT3 aggravated LPS-induced sepsis. (A)** After the last 4-OHT injection, *Rosa26^LSL-MLS-Stat3^* and *Rosa26^LSL-MLS-Stat3^;Ubc^ERT2Cre/+^* mice were intraperitoneally (i.p.) injected with LPS (20 mg/kg). Survival was monitored every 6 - 12 h after injection. The data shown represent a combination of two independent experiments. Statistical comparison of survival was performed with a log-rank test. **, *P* < 0.01. **(B)** Lung tissues were obtained from *Rosa26^LSL-MLS-Stat3^* (n = 3) and *Rosa26^LSL-MLS-Stat3^;Ubc^ERT2Cre/+^* mice (n = 3) injected with LPS for 24 h. One representative H&E staining photo per group is shown. Scale bar equals 100 µm. Serum LDH levels **(C)** and the levels of serum albumin **(D)** were assessed. The data shown are representative of one of two independent experiments and were compared using an unpaired Student's t-test. ** *P* < 0.01, * *P* < 0.05, n.s., not significant. **(E)** Serum samples were obtained from *Rosa26^LSL-MLS-Stat3^* (n = 3) and *Rosa26^LSL-MLS-Stat3^;Ubc^ERT2Cre/+^* mice (n = 3) after the treatment with LPS for 1 h and subjected to a cytokine array analysis. **(F-G)** Serum samples were obtained from *Rosa26^LSL-MLS-Stat3^* (n = 3) and *Rosa26^LSL-MLS-Stat3^;Ubc^ERT2Cre/+^* mice (n = 3) injected with LPS at the indicated time points and subjected to a cytometric bead array (CBA) analysis of the concentration of cytokines. The data shown are representative of one of two independent experiments. Numerical data are shown as the mean ± SD and were compared using two-way ANOVA followed by multiple comparisons. * *P* < 0.05, n.s., not significant. **(H)** Immunofluorescence of CD68 (red) and DAPI (blue) in lung tissue sections from LPS-induced sepsis. The data are representative of one of two independent experiments. The number of CD68^+^ cells was counted in the lung sections (Left panel). The data are representative of one of two independent experiments. **(I)** Single cells were prepared, and the percentages of macrophages were analyzed by flow cytometry. The data shown represent a combination of two independent experiments. (H-I) Numerical data are shown as the mean ± SD and were compared using an unpaired Student's t-test. ** *P* < 0.01, n.s., not significant.

Since we and others have found that the phosphorylation of STAT3 on Ser727 in BMDMs treated with LPS was induced within 30 min, STAT3 was phosphorylated on the tyrosine 705 residue when LPS stimulation was prolonged to approximately 65 min (**Figure S2D**), suggesting that p-STAT3 (Try705) could be a secondary and indirect activation 31. To study the function of mitochondrial STAT3, we injected mice from the same littermate intraperitoneally with 4-OHT followed by LPS to induce a murine sepsis model. In contrast to our expectation, 21 of 22 *Rosa26^LSL-MLS-mSTAT3^*;*Ubc^ERT2Cre/+^* mice died within 72 h after being challenged with LPS, while 6 of 21 *Rosa26^LSL-MLS-mSTAT3^* mice survived more than 144 h (**Figure 2A**). The mitochondrial STAT3 knock-in mice developed much more severe septic pneumonia as indicated by more infiltrated immune cells (**Figure 2B**). The levels of LDH, which is closely related to lung injury 32, were significantly increased in the *Rosa26^LSL-MLS-mSTAT3^*;*Ubc^ERT2Cre/+^* mice compared with those in the *Rosa26^LSL-MLS-mSTAT3^* mice (**Figure 2C**). Lung injury has been indicated by vascular leakage of serum albumin 33. Therefore, we examined the serum albumin levels and found that the levels of albumin were significantly reduced in the *Rosa26^LSL-MLS-mSTAT3^*;*Ubc^ERT2Cre/+^* mice compared with those in the *Rosa26^LSL-MLS-mSTAT3^* mice (**Figure 2D**). The production of multiple cytokines, including IL-6 and IL-1β, was increased (**Figures 2E-G**). MCP-1 was released by resident macrophages 34. Here, we found that it was necessary to examine the number of infiltrated macrophages in lung tissue. To assess the infiltrated macrophages in the lung tissues, we examined the expression of CD68, a macrophage marker in lung tissues, by Immunofluorescence staining. The number of infiltrated macrophages was clearly increased in the lung tissues from the *Rosa26^LSL-MLS-mSTAT3^*;*Ubc^ERT2Cre/+^* mice compared with that in the *Rosa26^LSL-MLS-mSTAT3^* mice (**Figure 2H**). We also performed FACS to analyze the macrophages in the lung tissues and found that the percentages of Ly6G^-^CD64^+^F4/80^+^ macrophages, but not alveolar macrophages (Ly6G^-^CD64^+^F4/80^+^CD11c^+^CD11b^-^) were potently increased in the lung tissues from the *Rosa26^LSL-MLS-mSTAT3^*;*Ubc^ERT2Cre/+^* mice compared that in the tissues from the *Rosa26^LSL-MLS-mSTAT3^* mice (**Figure 2I**). These observations indicate that mitochondrial STAT3 could promote macrophage activation after LPS challenge.

### Mitochondrial STAT3 promoted macrophage activation

**Figure 3:**
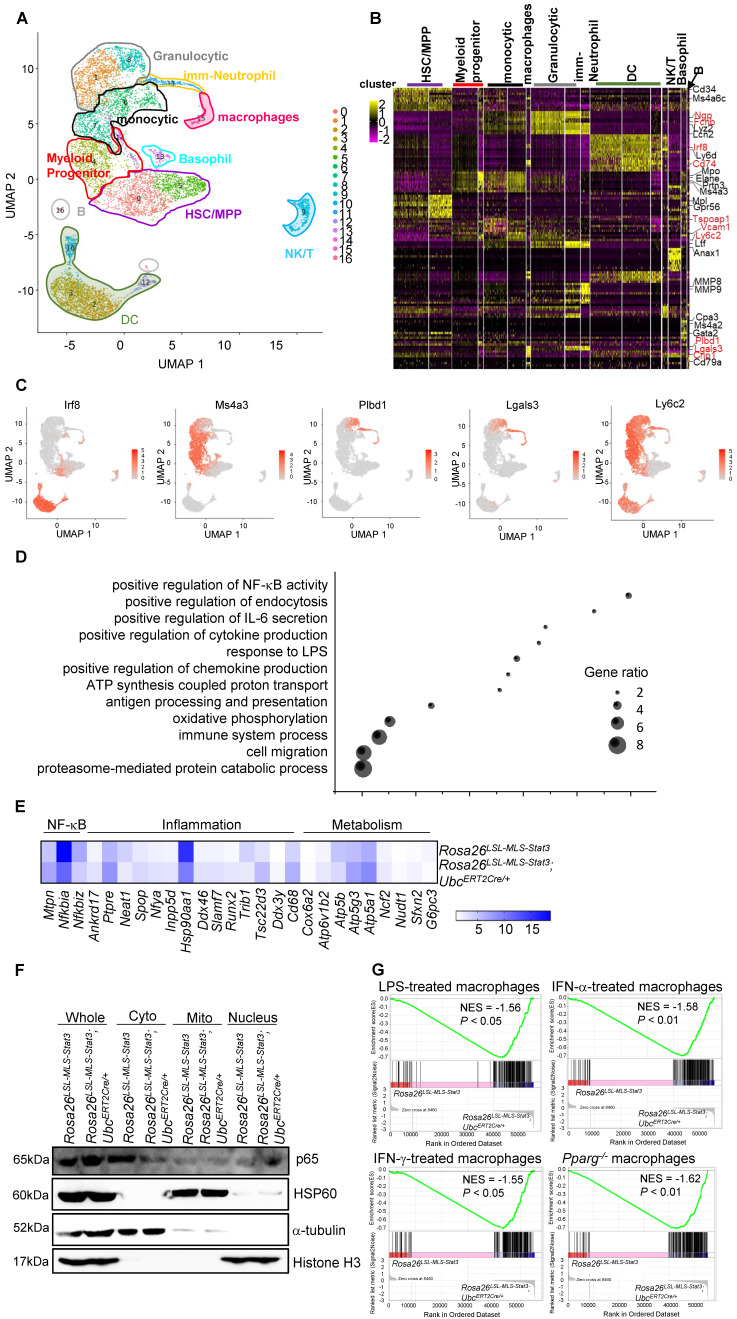
** Mitochondrial STAT3 promoted macrophage activation. (A)** Visualized t-SNE maps of c-Kit^+^ BM cells from *Rosa26^LSL-MLS-Stat3^* (n = 3) and *Rosa26^LSL-MLS-Stat3^;Ubc^ERT2Cre/+^* mice (n = 3) treated with 4-OHT. Phenograph clustering was performed, and the clusters were grouped into each myeloid lineage according to known lineage-restricted markers. **(B)** Heatmap showing the row-scaled expression of the 20 highest DEGs (Bonferroni-corrected *P* values < 0.05; Student's t-test) per cluster. Known key factors are indicated. Macrophage-specific genes are highlighted in red. **(C)** Expression of five typical macrophage genes. **(D)** Gene Ontology analysis of cluster (monocytes and macrophages)-based DEGs between the *Rosa26^LSL-MLS-Stat3^* mice and *Rosa26^LSL-MLS-Stat3^;Ubc^ERT2Cre/+^* mice. Selected Gene Ontology termed with |avg_logFC| > 0.25 and *P* values < 0.05. **(E)** Heatmap showing the expression of genes involved in NF-κB, inflammation and metabolism in macrophage clusters. Selected genes were termed |avg_logFC| > 0.25 and *P* values < 0.05. **(F)** BMDMs from *Rosa26^LSL-MLS-Stat3^* and *Rosa26^LSL-MLS-Stat3^;Ubc^ERT2Cre/+^* cells were induced. Cells were treated with 100 nM 4-OHT for 3 days. Nuclear, cytoplasmic, and mitochondrial fractions were extracted. The levels of the indicated proteins were analyzed by a Western blot analysis. The data shown are representative of one of three independent experiments. **(G)** GSEA analyses of gene sets of macrophage activation. NES, normalized enrichment score. Negative NES indicates higher expression in macrophages from *Rosa26^LSL-MLS-Stat3^;Ubc^ERT2Cre/+^* mice.

Macrophages are derived from monocytes, which constitute a heterogeneous cell type consisting of phenotypically and functionally distinct subpopulations 35. To examine whether activated macrophages originated from mitochondrial STAT3-derived potential monocytic populations, we sorted c-Kit^+^ cells and performed a single-cell analysis. Unbiased, graph-based clustering identified 16 major cell populations (**Figure 3A**). Based on the current literature 36-40, we could partition the cell identities in our dataset into ten clusters (**Figure 3A**). The monocytic clusters and macrophage clusters possessed key monocytic genes, such as *Irf8*, and *Ms4a3*, a specific marker of BM GMP and cMoP stages 41, *Plbd1* 42 and *Lgals3* 43 (**Figures 3B-C, Figure S3A**). The analysis revealed that many genes enriched in monocytes and macrophages were enriched in their progenitors and neutrophil granules (myeloid progenitors,** Figure 3B**).

A deeper analysis was performed to investigate whether mitochondrial STAT3 affected the gene expression involved in mediating monocytic cell function. The GO analysis demonstrated that mitochondrial STAT3 in monocytic cells and macrophages triggered a significant upregulation of genes involved in immune system processes, response to lipopolysaccharide, and positive regulation of NF-κB transcription factor activity (**Figure 3D**). Furthermore, genes related to metabolism, such as *Atp5a1* and *Atp5g3*, and inflammation, such as *Tsc22d3,* were upregulated (**Figure 3E, Figure S3B**).

Meanwhile, genes involved in suppressing the immune response, such as *Hsp90aa1* were downregulated (**Figure 3E, Figure S3B**). Interestingly, the expression of the *Nfkbia* and *Nfkniz* genes encoding inhibitors of NF-κB was decreased in these macrophages (**Figure 3E, Figure S3B**), while the expression of the *Mtpn* gene-encoding protein, which plays an important role in mediating NF-κB activation, was elevated in the macrophages from the *Rosa26^LSL-MLS-mSTAT3^*;*Ubc^ERT2Cre/+^* mice (**Figure 3E, Figure S3B**). Consistently, the levels of nuclear NF-κB in the *Rosa26^LSL-MLS-mSTAT3^*;*Ubc^ERT2Cre/+^* macrophages were higher than those in the *Rosa26^LSL-MLS-mSTAT3^* macrophages (**Figure 3F, Figure S3C**).

We performed gene expression profiling and a gene set enrichment analysis (GSEA) to further determine whether mitochondrial STAT3 triggered macrophage activation. The GSEA revealed a correlation between the upregulated genes in the mitochondrial STAT3 knock-in macrophages and the MSigDB immunological process gene signatures associated with LPS-treated macrophages, IFN-α-treated macrophages, and IFN-γ-treated macrophages (**Figure 3G**). These data suggest that when STAT3 was localized in mitochondria, genes related to metabolic reprogramming and immune response were upregulated, leading to macrophage activation, although the subpopulation of macrophages could be maintained.

### Mitochondrial STAT3 influenced the energy source switch in septic mice

**Figure 4:**
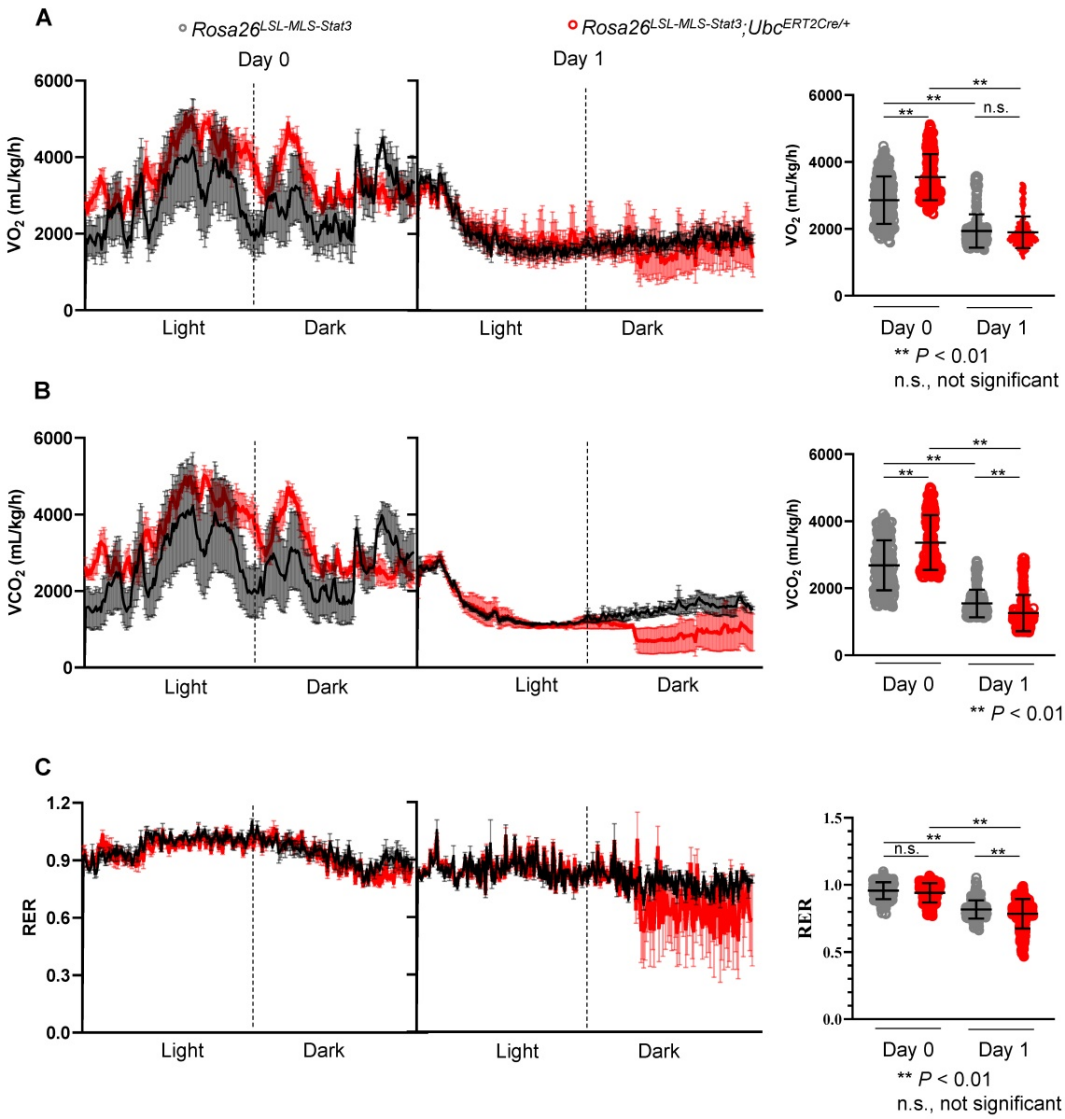
** Mitochondrial STAT3 influenced the energy source switch in septic mice.** *Rosa26^LSL-MLS-Stat3^* and *Rosa26^LSL-MLS-Stat3^;Ubc^ERT2Cre/+^* mice were treated with 4-OHT 7 times, followed by LPS injection. **(A)** O_2_ consumption rates 24 h before (Day 0) and after (Day 1) LPS injection were measured by indirect calorimetry using CLAMS under chow-fed conditions, and the average O_2_ consumption rate is shown on the right (n = 4 mice/group). **(B)** CO_2_ production rates 24 h before (Day 0) and after (Day 1) LPS injection were measured by indirect calorimetry using CLAMS under chow-fed conditions, and the average CO_2_ production rate is shown on the right (n = 4 mice/group). **(C)** The respiratory exchange ratio 24 h before (Day 0) and after (Day 1) LPS injection was measured by indirect calorimetry using CLAMS under chow-fed conditions. Histogram representing the average RER (n = 4 mice/group). (A-C) Significance was calculated using a two-way ANOVA, followed by multiple comparisons test. **, *P* < 0.01, n.s., not significant.

It is known that metabolic programming can trigger the gene regulatory circuits that control complex cell behaviors 44. Here, to determine whether energy source switching occurred in the LPS-treated *Rosa26^LSL-MLS-mSTAT3^*;*Ubc^ERT2Cre/+^* mice, we first analyzed the metabolic differences between the *Rosa26^LSL-MLS-mSTAT3^*;*Ubc^ERT2Cre/+^* mice and *Rosa26^LSL-MLS-mSTAT3^* mice *in vivo*. The results showed that the *Rosa26^LSL-MLS-mSTAT3^*;*Ubc^ERT2Cre/+^* mice exhibited a significant increase in VO_2_ in parallel with elevated VCO_2_ compared to the *Rosa26^LSL-MLS-mSTAT3^* mice (**Figures 4A-B**, Day 0 panel). However, the respiratory exchange ratio (RER), which is the ratio of VCO_2_/VO_2_ used to refer to the energy source 45, was almost identical (**Figure 4C**, Day 0 panel). After the LPS challenge, there was no difference between the *Rosa26^LSL-MLS-mSTAT3^*;*Ubc^ERT2Cre/+^* mice and *Rosa26^LSL-MLS-mSTAT3^* mice, although the levels of VO_2_ were dramatically reduced in these two groups of mice (**Figure 4A**, Day 1 panel). Interestingly, upon the LPS stimulation, the levels of VCO_2_ were obviously decreased in the *Rosa26^LSL-MLS-mSTAT3^*;*Ubc^ERT2Cre/+^* mice (**Figure 4B**, Day 1 panel). Additionally, when challenged with LPS, the RER value in the *Rosa26^LSL-MLS-mSTAT3^*;*Ubc^ERT2Cre/+^* mice (0.76 ± 0.09) was lower than that in the *Rosa26^LSL-MLS-mSTAT3^* mice (0.81 ± 0.07) (**Figure 4C**, Day 1 panel). These observations suggest that *in vivo*, after being challenged by LPS, mitochondrial STAT3 could promote a switch from glucose to lipid substrates as the energy source, at least partially.

### Mitochondrial STAT3 enhanced FAO in LPS-treated macrophages

**Figure 5:**
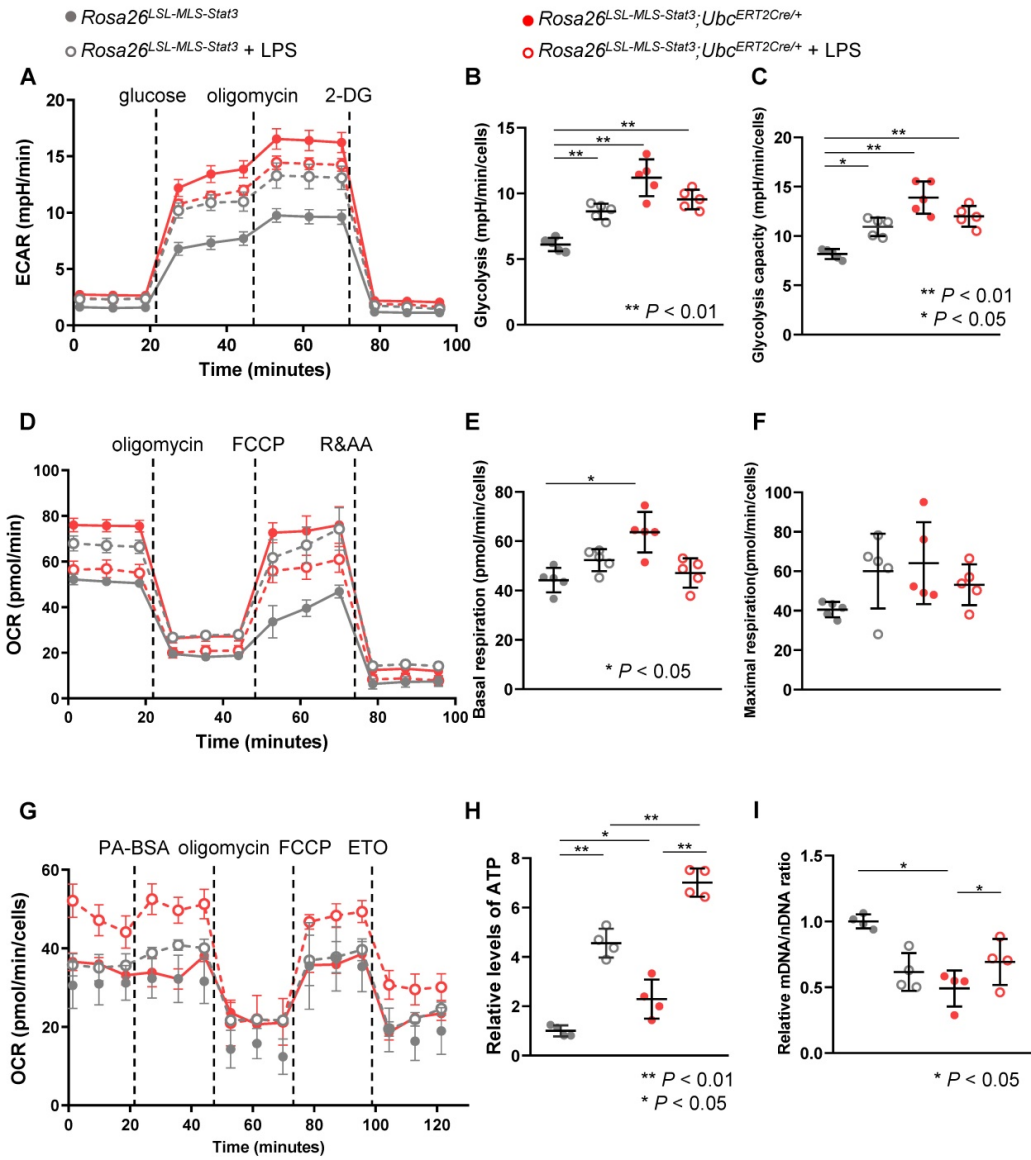
** Mitochondrial STAT3 enhanced FAO in LPS-treated macrophages. (A)** BMDMs of *Rosa26^LSL-MLS-Stat3^* and *Rosa26^LSL-MLS-Stat3^;Ubc^ERT2Cre/+^* mice were induced and treated with 100 nM 4-OHT for another 3 days. Then, these cells were stimulated with or without 100 ng/mL LPS for 6 h. The ECAR of the BMDMs was measured at baseline and after activation with LPS in a Seahorse extracellular flux analyzer (EFA). For ECAR, the cells were treated by sequential injection of the following compounds: glucose (30 mmol/L), oligomycin (2 μmol/L), and 2-deoxyglucose (2-DG, 100 mmol/L). **(B)** Quantification of glycolysis in (A). **(C)** Quantification of glycolysis capacity in (A). **(D)** After 4-OHT induction, BMDMs were stimulated with or without 100 ng/mL LPS for 6 h. The BMDMs were treated by sequential injection of the following compounds: oligomycin (2 μmol/L), carbonyl cyanide-4 (trifluoromethoxy) phenylhydrazone (FCCP, 4 μmol/L), and antimycin A (1 μmol/L) plus rotenone (100 nmol/L). **(E)** Quantification of basal respiration in (D). **(F)** Quantification of maximal respiration in (D). (B and C, E and F) Data are shown as the mean ± SD and are representative of one of two independent experiments. Significance was calculated using a two-way ANOVA, followed by multiple comparisons test. **, *P* < 0.01, *, *P* < 0.05. **(G)** Long-term extracellular flux analysis (EFA) of FAO substrate OCRs. *Rosa26^LSL-MLS-Stat3^* and *Rosa26^LSL-MLS-Stat3^;Ubc^ERT2Cre/+^* BMDMs were induced and treated with 100 nM 4-OHT for another 3 days. Then, these cells were stimulated with or without 100 ng/mL LPS for 6 h before loading. BMDMs were then treated sequentially with BSA-conjugated palmitate, oligomycin, FCCP, and ETO as indicated. The data are representative of one of two independent experiments performed with n = 5. **(H)** Lysates of BMDMs treated with or without 100 ng/mL LPS were prepared and subjected to measure the ATP levels. **(I)** RNA was extracted from BMDMs treated with or without 100 ng/mL LPS and subjected to real-time RT-PCR. (H and I) Data are shown as the mean ± SD and are representative of one of two independent experiments performed with n = 4. Significance was calculated using a two-way ANOVA, followed by multiple comparisons test. **, *P* < 0.01, *, *P* < 0.05.

During this phase, we performed seahorse experiments to determine whether a similar energy source shift occurred. Without the LPS treatment, glycolysis and the glycolytic capacity were significantly increased in the *Rosa26^LSL-MLS-mSTAT3^*;*Ubc^ERT2Cre/+^* macrophages (**Figures 5A-C**). Unexpectedly, in the presence of LPS, glycolysis and the glycolytic capacity in the *Rosa26^LSL-MLS-mSTAT3^*;*Ubc^ERT2Cre/+^* macrophages were reduced compared to those in the control diluent-treated *Rosa26^LSL-MLS-mSTAT3^*;*Ubc^ERT2Cre/+^* macrophages (**Figures 5A-C**).

The *Rosa26^LSL-MLS-mSTAT3^*; *Ubc^ERT2Cre/+^* macrophages showed higher basal and maximal OCRs (**Figures 5D-F**). After the LPS challenge, the basal and maximal oxidative capacity was slightly increased in the *Rosa26^LSL-MLS-mSTAT3^* macrophages (**Figures 5D-F**), which could be related to the relatively long culture conditions. However, in the presence of LPS, the basal and maximal oxidative capacity was decreased in the *Rosa26^LSL-MLS-mSTAT3^*; *Ubc^ERT2Cre/+^* macrophages (**Figures 5D-F**). Interestingly, the LPS-treated *Rosa26^LSL-MLS-mSTAT3^*; *Ubc^ERT2Cre/+^* macrophages showed an increase in OCR when cultured in medium containing BSA-conjugated palmitic acid (**Figure 5G**).

Notably, this sustained OCR precipitously decreased in response to etomoxir (**Figure 5G**), indicating that in the presence of LPS, to compensate for the reduced energy production via glucose utilization, mitochondrial STAT3 endowed macrophages with higher levels of FAO for the production of ATP. Indeed, ATP production in the *Rosa26^LSL-MLS-mSTAT3^*; *Ubc^ERT2Cre/+^* macrophages was significantly elevated compared to that in the *Rosa26^LSL-MLS-mSTAT3^* macrophages (**Figure 5H**). Moreover, ATP production in the LPS-treated *Rosa26^LSL-MLS-mSTAT3^*; *Ubc^ERT2Cre/+^* macrophages was dramatically higher than that in their parental control cells and LPS-treated *Rosa26^LSL-MLS-mSTAT3^* macrophages (**Figure 5H**).

The ratio of mitochondrial DNA to nuclear DNA (mtDNA/nDNA) was quantified to examine whether mitochondrial STAT3 induced mitochondrial biogenesis, and we found that in contrast to the LPS-treated *Rosa26^LSL-MLS-mSTAT3^*; *Ubc^ERT2Cre/+^* macrophages, the ratio was reduced in the LPS-treated *Rosa26^LSL-MLS-mSTAT3^* macrophages (**Figure 5I**). These observations show that in the presence of LPS, mitochondrial STAT3 led to an imbalance in mitochondrial metabolism and promoted mitochondrial biogenesis in which increased FAO compensated for the energy source.

### Mitochondrial STAT3 facilitated CPT1a stabilization

**Figure 6:**
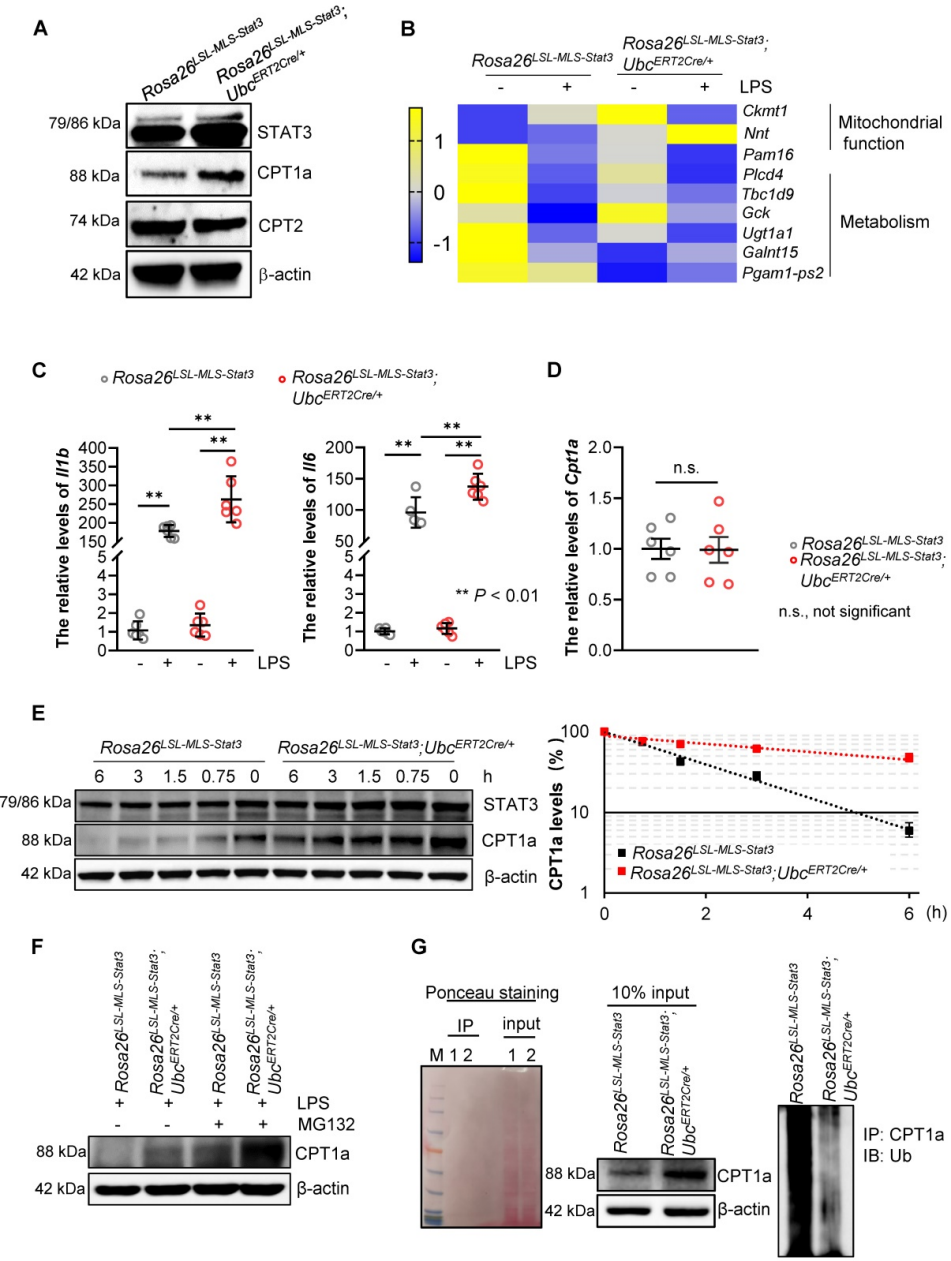
** Mitochondrial STAT3-driven CPT1a stabilization in LPS-treated macrophages. (A)** Lysates were extracted from BMDMs of *Rosa26^LSL-MLS-Stat3^* and *Rosa26^LSL-MLS-Stat3^;Ubc^ERT2Cre/+^* mice and treated with LPS for 6 h. The levels of the indicated proteins were analyzed by a Western blot analysis. The data shown are representative of one of three independent experiments. **(B)** RNA was extracted from BMDMs treated with or without LPS and subjected to gene profiling. Heatmap showing the row-scaled expression of the highest DEGs. **(C)** RNA was extracted from BMDMs treated with or without 100 ng/mL LPS and subjected to real-time RT-PCR. Data are shown as the mean ± SD and represent one of two independent experiments performed in duplicate (n = 3). Significance was calculated using a two-way ANOVA, followed by multiple comparisons test. **, *P* < 0.01.** (D)** RNA was extracted from LPS-treated BMDMs (n = 3) and subjected to real-time RT-PCR. Data are shown as the mean ± SD and represent one of two independent experiments performed in duplicate. Significance was compared using an unpaired Student's t-test. n.s., not significant.** (E)** BMDMs of the *Rosa26^LSL-MLS-Stat3^* and *Rosa26^LSL-MLS-Stat3^;Ubc^ERT2Cre/+^* genotypes were treated with 4-OHT for 3 days. Then, these cells were treated with 100 ng/mL LPS for another 6 h in the presence of 100 μg/mL cycloheximide and harvested at the indicated time points. STAT3, CPT1a, and actin were analyzed by a Western blot analysis. The relative levels of CPT1a were quantified, normalized to actin, and plotted. The figure (left panel) shown are representative of one of two independent experiments. The data (right panel) shown represent a combination of two independent experiments. **(F)** BMDMs of the indicated genotypes were treated with 4-OHT for 3 days. Then, these cells were treated with 100 ng/mL LPS and 10 μmol/L MG132 for an additional 6 h. Cell lysates were isolated, and the levels of CPT1a and actin were analyzed by a Western blot analysis. **(G)** BMDMs of the indicated genotypes were treated with 4-OHT for 3 days. Then, these cells were treated with 100 ng/mL LPS and 10 μmol/L MG132 for an additional 6 h before harvesting. The cell lysates were isolated, IP with an anti-CPT1a antibody and analyzed by a Western blot analysis using an anti-ubiquitin antibody. 1, *Rosa26^LSL-MLS-Stat3^*; 2, *Rosa26^LSL-MLS-Stat3^;Ubc^ERT2Cre/+^*. (E and F) The data shown represent one of three independent experiments.

To investigate the mechanisms by which mitochondrial STAT3 enhanced FAO in the LPS-treated macrophages, we examined the expression of CPT1a, the rate-limiting enzyme of FAO. The results demonstrated that the expression of CPT1a, but not CPT2, was noticeably elevated in the *Rosa26^LSL-MLS-mSTAT3^*; *Ubc^ERT2Cre/+^* macrophages (**Figure 6A, Figure S4A**). Interestingly, the number of genes involved in mitochondrial function and inflammation, including *Il1b* and *Il6,* was increased in the *Rosa26^LSL-MLS-mSTAT3^*; *Ubc^ERT2Cre/+^* macrophages in the presence of LPS compared with that in their parental cells and *Rosa26^LSL-MLS-mSTAT3^* macrophages treated with or without LPS (**Figures 6B-C**). However, upon the LPS treatment, the mRNA levels of *Cpt1a* were almost identical in these macrophages (**Figure 6D**), indicating that CPT1a protein accumulation could be caused by posttranslational modification.

To confirm that CPT1a protein accumulation is affected by mitochondrial STAT3 in the presence of LPS, we performed a protein half-life assay. CPT1a exhibited a relatively short half-life of approximately 90 min in the LPS-treated* Rosa26^LSL-MLS-mSTAT3^* macrophages (**Figure 6E**). In contrast, in the LPS-treated *Rosa26^LSL-MLS-mSTAT3^*; *Ubc^ERT2Cre/+^* macrophages, CPT1a degradation was largely inhibited with a half-life of approximately 6 h (**Figure 6E**). To investigate whether the extended half-life of CPT1a is due to reduced CPT1a proteasomal degradation in LPS-treated *Rosa26^LSL-MLS-mSTAT3^*; *Ubc^ERT2Cre/+^* macrophages, we exposed LPS-treated macrophages to the proteasome inhibitor MG-132. Following the administration of MG-132, the expression of CPT1a was increased in the LPS-treated *Rosa26^LSL-MLS-mSTAT3^*; *Ubc^ERT2Cre/+^* macrophages (**Figure 6F, Figure S4B**). Additionally, to determine whether CPT1a ubiquitination is impaired in LPS-treated *Rosa26^LSL-MLS-mSTAT3^*; *Ubc^ERT2Cre/+^* macrophages, we performed a ubiquitination assay, and Ponceau S staining was utilized to demonstrate that the same amount of protein was loaded in SDS-PAGE. High molecular weight smears, which are indicative of polyubiquitinated CPT1a protein species, were observed in the LPS-treated *Rosa26^LSL-MLS-mSTAT3^* macrophages, but not the LPS-treated *Rosa26^LSL-MLS-mSTAT3^*; *Ubc^ERT2Cre/+^* macrophages (**Figure 6G**). These data suggest that mitochondrial STAT3 could play a vital role in mediating CPT1a polyubiquitination and proteasomal degradation in LPS-treated macrophages.

### Mitochondrial STAT3 driven CPT1a-mediated FAO via USP50

**Figure 7:**
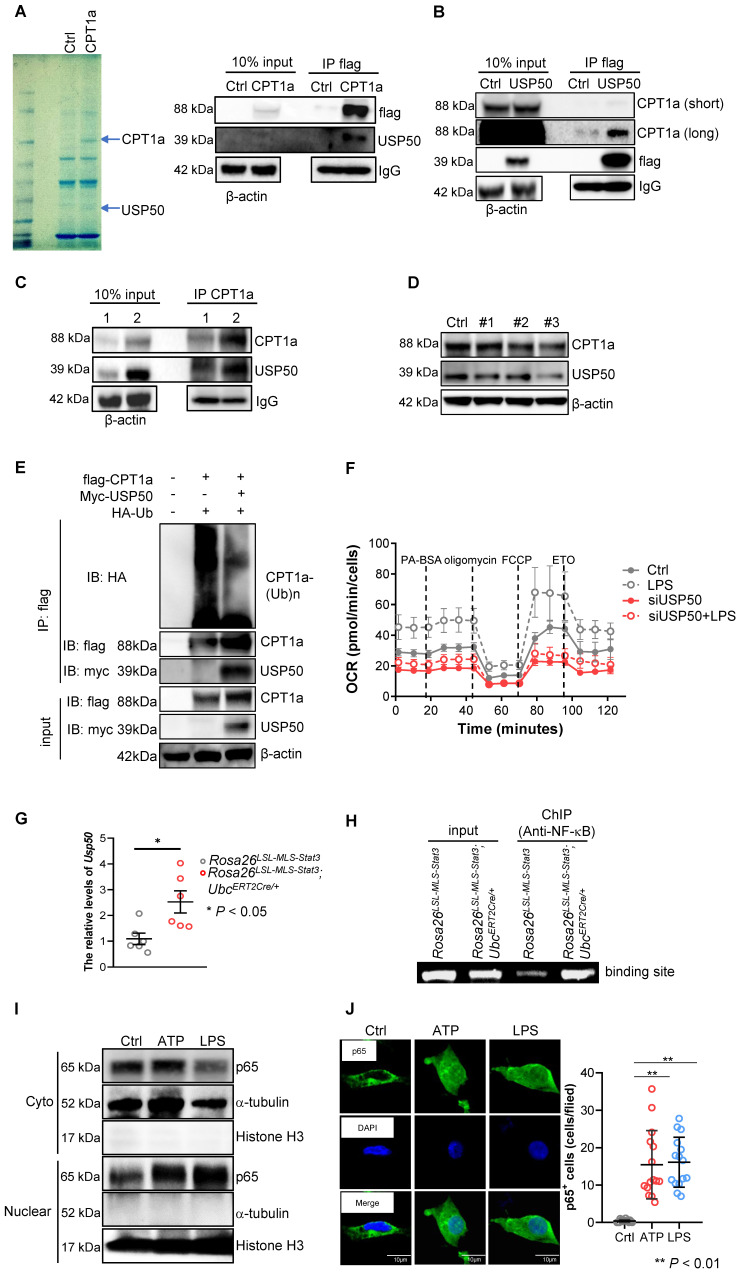
** Mitochondrial STAT3 driven CPT1a-mediated FAO via USP50. (A)** CPT1a complexes were purified from 293T cells overexpressing either empty (Ctrl) or CPT1a tagged at the C-terminus with a flag epitope (CPT1a). The complexes were analyzed by mass spectrometry and a Western blot analysis. **(B)** USP50 complexes were purified from 293T cells overexpressing either empty (Ctrl) or USP50-flag (USP50). The complexes were analyzed by a Western blot analysis. **(C)** BMDMs of the indicated genotypes were treated with 4-OHT for 3 days, followed by treatment with 100 ng/mL LPS for an additional 6 h before harvesting. The cells were harvested, lysed, IP for CPT1a, and blotted with the indicated proteins. The input represents 10% of the total cell lysate utilized for IP. 1, *Rosa26^LSL-MLS-Stat3^*; 2, *Rosa26^LSL-MLS-Stat3^;Ubc^ERT2Cre/+^*. **(D)** Western blot analysis. *Rosa26^LSL-MLS-Stat3^* macrophages were transfected with a negative control siRNA (Ctrl) or a siRNAs against USP50. After 24 h, proteins were extracted and subjected to a Western blot analysis. The membrane was sequentially probed with the indicated antibodies. The results shown are representative of one of three independent experiments. **(E)** 293T cells were transfected with the indicated plasmid DNA. After 24 h, the lysates were harvested and incubated with anti-Flag beads overnight, followed by a Western blot analysis. **(F)** Long-term extracellular flux analysis (EFA) of FAO substrate OCRs. *Rosa26^LSL-MLS-Stat3^* and *Rosa26^LSL-MLS-Stat3^;Ubc^ERT2Cre/+^* BMDMs were induced and treated with 100 nM 4-OHT for another 3 days. Then, these cells were transfected with a negative control siRNA or a siRNA against USP50. After 24 h, these cells were stimulated with or without 100 ng/mL LPS for 6 h before loading. BMDMs were then treated sequentially with BSA-conjugated palmitate, oligomycin, FCCP, and ETO as indicated. The data are representative of one of two independent experiments performed with n = 5. **(G)** BMDMs (n = 3) were induced and treated with 100 nM 4-OHT for another 3 days. Then, these cells were stimulated with or without 100 ng/mL LPS for 6 h, followed by real-time RT-PCR. The data are shown as the mean ± SD and are representative of one of two independent experiments performed in duplicate (n = 3). Significance was calculated using a student's t-test. *, *P* < 0.05.** (H)** BMDMs were induced, treated with 100 nM 4-OHT for another 3 days, and exposed to 100 ng/mL LPS for 6 h. After being crosslinked, the protein-DNA complexes were immunoprecipitated using an anti-NF-κB antibody. Then, DNA was extracted and subjected to PCR to measure the NF-κB binding sites on the USP50 promoter. The PCR products were separated on a 2% agarose gel and visualized by ethidium bromide staining. The data shown are representative of one of two independent experiments with similar results.** (I)** *Rosa26^LSL-MLS-Stat3^* BMDMs were treated with 5 μmol/L ATP or 100 ng/mL LPS for 15 min before harvesting. Nucleus and cytoplasmic fractions were extracted. The levels of the indicated proteins were analyzed by a Western blot analysis. **(J)** *Rosa26^LSL-MLS-Stat3^* BMDMs were treated with 5 μmol/L ATP or 100 ng/mL LPS for 15 min. The cells were fixed, permeabilized, stained with anti-p65 antibody, and counterstained with DAPI, and the cell images were taken under a microscope. Representative images are shown, and p65-positve cells were counted.

To identify the proteins involved in blocking CPT1a proteasomal degradation mediated by mitochondrial STAT3, we first performed IP coupled with LC-MS and revealed that USP50, a deubiquitinating enzyme, could interact with CPT1a (**Figure 7A**). Additionally, CPT1a interacted with USP50 in 293T cells transfected with flag-USP50 (**Figure 7B**). Importantly, the IP of CPT1a pulled down a significant amount of USP50 in the LPS-treated *Rosa26^LSL-MLS-mSTAT3^*; *Ubc^ERT2Cre/+^* macrophage lysates (**Figure 7C**, IP Lane 2**, Figure S5A**). In comparison, in the LPS-treated *Rosa26^LSL-MLS-mSTAT3^* macrophage lysates, CPT1a pulled down much less USP50 (**Figure 7C**, IP Lane 1**, Figure S5A**), suggesting that mitochondrial STAT3 could contribute to CPT1a stabilization by influencing CPT1a-USP50 binding, at least partially.

To determine whether UPS50 is a key mediator of mitochondrial STAT3-triggered FAO, we knocked down USP50 by siRNAs and found that the levels of UPS50 were decreased along with CPT1a, especially in #3 siRNA (**Figure 7D, Figure S5B**). To explore whether USP50 is critical for mediating CPT1a polyubiquitination, we performed IP and found that polyubiquitinated CPT1a protein species were observed in 293T cells transfected with flag-CPT1a and HA-ubiquitin (**Figure 7E**). However, the levels of polyubiquitinated CPT1a protein were decreased in the 293T cells transfected with flag-CPT1a, myc-USP50 and HA-ubiquitin (**Figure 7E**). Furthermore, silencing USP50 suppressed the OCR (**Figure 7F**). These results suggest that USP50 is a key factor contributing to mitochondrial STAT3-triggered FAO.

### USP50 was induced by NF-κB in LPS-treated mitochondrial STAT3 macrophages

To uncover the mechanisms by which mitochondrial STAT3 influenced the expression of USP50, we examined the mRNA levels of USP50 and found that the levels of USP50 mRNA were dramatically increased in the LPS-treated *Rosa26^LSL-MLS-mSTAT3^*; *Ubc^ERT2Cre/+^* macrophages (**Figure 7G**). Next, we predicted the binding sites of transcription factors on the USP50 promoter. As shown in Figure **7H**, NF-κB bound the UPS50 promoter. Studies have shown that LPS/ATP-induced inflammation is predominantly controlled by NF-κB 46.

The ATP levels were higher in the *Rosa26^LSL-MLS-mSTAT3^*; *Ubc^ERT2Cre/+^* macrophages (**Figure 5H**). Here, we sought to examine whether ATP stimulated NF-κB nuclear localization. In the presence of ATP, the NF-κB levels in the nucleus were higher (**Figure 7I, Figure S5C**). In addition, ATP alone induced NF-κB nuclear localization (**Figure 7J**). These observations suggest that mitochondrial STAT3 facilitated NF-κB nuclear localization by elevating ATP, leading to USP50 expression.

### Curcumin relieved LPS-induced sepsis by suppressing the activation of STAT3 and NF-κB

**Figure 8:**
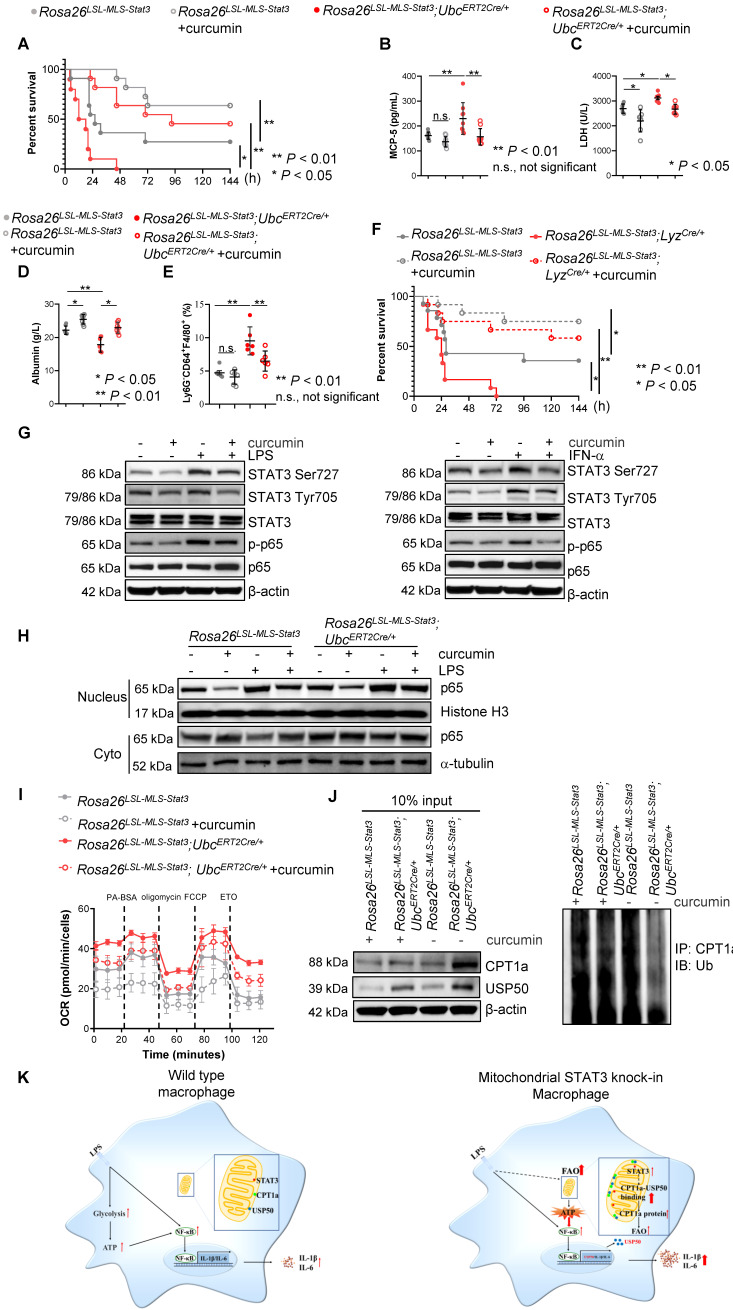
** Curcumin relieved LPS-induced sepsis. (A)** After the last 4-OHT injection, *Rosa26^LSL-MLS-Stat3^* and *Rosa26^LSL-MLS-Stat3^;Ubc^ERT2Cre/+^* mice were injected with curcumin prior to LPS. Survival was monitored every 6 - 12 h after injection. The data shown represent a combination of two independent experiments. Statistical comparison of survival was performed with a log-rank test. **, *P* < 0.01, *, *P* < 0.05. **(B)** Serum samples were obtained from these mice and subjected to ELISA to examine MCP-5 levels. **(C-D)** The serum levels of LDH and albumin were assessed. **(E)** Lung tissues were removed, and the percentages of macrophages were analyzed by flow cytometry. (B-E) The data are representative of one of two independent experiments. Significance was calculated using a two-way ANOVA. **, *P* < 0.01, * *P* < 0.05, n.s., not significant. **(F)** *Rosa26^LSL-MLS-Stat3^* and *Rosa26^LSL-MLS-Stat3^;Lyz^Cre/+^* mice were injected with 10 mg/kg curcumin prior to LPS (20 mg/kg). Survival was monitored every 6 - 12 h after injection. Statistical comparison of survival was performed with a log-rank test. **, *P* < 0.01, *, *P* < 0.05. **(G)** BMDMs of the indicated genotypes were treated with 8 μM curcumin for 20 h and incubated with either 100 ng/mL LPS (30 min) or 100 ng/mL IFN-α (1 h) before harvesting. The cells were harvested, lysed, and subjected to a Western blot analysis. The membranes were blotted with the indicated antibodies. **(H)** Cells were treated with 8 μM curcumin for 20 h and incubated with 100 ng/mL LPS for another 15 min. Nuclei and cytoplasmic fractions were extracted. The levels of the indicated proteins were analyzed by a Western blot analysis. **(I)** BMDMs were treated with curcumin. After 20 h, these cells were stimulated with or without 100 ng/mL LPS for 6 h before loading. BMDMs were then treated sequentially with BSA-conjugated palmitate, oligomycin, FCCP, and ETO as indicated. The data are representative of one of two independent experiments performed with n = 5. **(J)** 4-OHT-treated BMDMs were treated with curcumin for 20 h, and then, the BMDMs were exposed to 100 ng/mL LPS and 10 μmol/L MG132 for an additional 6 h before harvesting. The cell lysates were isolated, IP with an anti-CPT1a antibody and analyzed by a Western blot analysis using an anti-ubiquitin antibody. (G, H and J) The results shown are representative of one of three independent experiments.** (K)** Schematic representation of the mitochondrial STAT3-mediated increase in FAO in a USP50-dependent manner in the LPS-induced sepsis model. Upon the LPS stimulation, mitochondrial STAT3 could induce CPT1a stabilization by promoting USP50 expression and facilitating CPT1a-USP50 binding, leading to a switch from glucose to an increased reliance on FAO for ATP production, which, in turn, further enhanced NF-κB nuclear localization, leading to a great amount of cytokine production.

Curcumin is a well-documented herbal anti-inflammatory agent with low toxicity and few adverse reactions 47 that has been shown to inhibit nuclear STAT3 function and NF-κB activity in tumors 48, 49. We speculated that curcumin could alleviate the severity of LPS-induced sepsis, at least partially, by decreasing NF-κB activity. As expected, curcumin extended the survival curve of the LPS-treated *Rosa26^LSL-MLS-mSTAT3^*; *Ubc^ERT2Cre/+^* mice and *Rosa26^LSL-MLS-mSTAT3^* mice in parallel with a decrease in the levels of MCP-5 that's primarily produced by activated macrophages 50(**Figures 8A-B**). In addition, upon the curcumin treatment, the serum levels of LDH were diminished in the LPS-treated *Rosa26^LSL-MLS-mSTAT3^*; *Ubc^ERT2Cre/+^* mice and *Rosa26^LSL-MLS-mSTAT3^* mice (**Figure 8C**). In the presence of curcumin, the levels of albumin were elevated in the LPS-treated *Rosa26^LSL-MLS-mSTAT3^*; *Ubc^ERT2Cre/+^* mice and *Rosa26^LSL-MLS-mSTAT3^* mice (**Figure 8D**). Moreover, the percentage of Ly6G^-^CD64^+^F4/80^+^ macrophages was reduced in the lungs of the LPS-treated *Rosa26^LSL-MLS-mSTAT3^*; *Ubc^ERT2Cre/+^* mice and *Rosa26^LSL-MLS-mSTAT3^* mice as measured by FACS (**Figure 8E**), demonstrating that curcumin reversed macrophage turnover.

Macrophage dysfunction is a core determinant of sepsis development. To further confirm that the anti-sepsis effects of curcumin *in vivo* were primarily due to the inhibition of mitochondrial STAT3 knock-in macrophages by curcumin, we intercrossed *Rosa26^LSL-MLS-mSTAT3^* mice with *Lyz^Cre/+^* mice and treated the LPS-induced septic mice with either control diluent or curcumin. Consistent with the above data, all *Rosa26^LSL-MLS-mSTAT3^*; *Lyz^Cre/+^* mice died within 72 h (**Figure 8F**), while 5 of the 14* Rosa26^LSL-MLS-mSTAT3^* mice survived for as long as 144 h (**Figure 8F**). Following the curcumin treatment, 7 LPS-treated *Rosa26^LSL-MLS-mSTAT3^*; *Lyz^Cre/+^* mice (n=12) and 9 LPS-treated *Rosa26^LSL-MLS-mSTAT3^* mice (n=12) were alive (**Figure 8F**). Additionally, the levels of LDH and albumin were attenuated in the presence of curcumin in the LPS-induced sepsis mouse model (**Figures S6A-B**). These data indicate that macrophages harboring higher amounts of mitochondrial STAT3 worsened sepsis development. Curcumin protected these mice from LPS-mediated sepsis by influencing mitochondrial STAT3 function in macrophages.

To further investigate the mechanisms by which curcumin alleviated LPS-induced sepsis, we treated *Rosa26^LSL-MLS-mSTAT3^*; *Ubc^ERT2Cre/+^* macrophages with curcumin in the presence of either LPS or IFN-α. Consistently, the exposure of those macrophages to LPS (30 min) or IFN-α (60 min) and LPS elevated the levels of p-STAT3 (Ser727), but not p-STAT3 (Tyr705); INF-α increased the levels of both p-STAT3 (Ser727) and p-STAT3 (Tyr705) (**Figure 8G, Figures S6C-S6D**). In the presence of curcumin, the phosphorylated forms of p-STAT3 (Tyr705) induced by IFN-α were prohibited (**Figure 8G, Figure S6D**). Interestingly, curcumin retained the elevated expression of p-STAT3 (Ser727) induced by either LPS or IFN-α along with the decrease in the levels of p-p65 (**Figure 8G, Figures S6C-S6D**). The elevated levels of nuclear p65 induced by LPS were reduced in the presence of curcumin (**Figure 8H, Figure S6E**). In addition, LPS-triggered FAO was decreased by the administration of curcumin in the mitochondrial STAT3 knock-in macrophages (**Figure 8I**). Moreover, polyubiquitinated CPT1a was restrained in the presence of curcumin (**Figure 8J, Figure S6F**). These observations suggest that curcumin not only inhibited nuclear transcription factor STAT3 activity and NF-κB function but also targeted mitochondrial STAT3 function.

## Discussion

A recent study showed that upon LPS treatment, STAT3 interacts directly with TRAF6 and TBK-1, leading to the phosphorylation of STAT3 at residue 727. Subsequently, phosphorylated STAT3 is translocated into mitochondria and plays a critical role in LPS-induced metabolic reprogramming and inflammatory cytokine production *in vitro* and *in vivo* 31. These observations suggest that under infection conditions, such as LPS-induced sepsis, mitochondrial STAT3 could be a key factor triggering the metabolic reprogramming required for the rapid increase in demand for biosynthetic precursors and increased energy 31. To confirm the hypothesis, we examined STAT3 localization in phagocytes from sepsis patients. Our study results indicate that a subset of STAT3 accumulated in the mitochondrial fraction of phagocytes from sepsis patients (**Figures 1A and B**). To demonstrate the role of mitochondrial STAT3 *in vivo*, we utilized an inducible mitochondrial STAT3 knock-in mouse model and found that the expression of p-STAT3 (Ser727) was higher in mitochondria (**Figure 1E**). An unexpected result was that mitochondrial STAT3 exacerbated LPS-induced sepsis and promoted macrophage activation and the switch from glycolysis to an increased reliance on FAO for ATP production (**Figures 2, 4 and 5**).

A previous study used a genetically engineered mouse model (STAT3 SA) in which STAT3 was unable to be phosphorylated at the S727 residue to determine the mechanisms by which TLR4 activation contributes to mitochondrial reprogramming 31. In the presence of LPS, glycolysis and proinflammatory production in the STAT3 SA mice were reduced compared with those in the WT mice, indicating that STAT3 Ser727 is crucial for TLR4-induced metabolic reprogramming and inflammation 31. However, due to defective ETC activity, both basal EACR and OCR in STAT3 SA cells are lower than those in WT cells, which could be an obstacle to further investigating the role and mechanisms of mitochondrial STAT3 in mediating metabolic reprogramming in macrophages. In contrast to STAT3 SA mice, in our inducible knock-in mice, mitochondrial STAT3 can be switched “on” and “off” by the administration of 4-OHT or withdrawal of 4-OHT, respectively, allowing us to explore the function and mechanisms of mitochondrial STAT3 in controlling macrophage metabolism under physiological conditions or various stimuli.

In contrast to previous studies that demonstrated that macrophage activation by LPS was more reliant on aerobic glycolysis 51, glycolysis and the glycolysis capacity were reduced in the *Rosa26^LSL-MLS-mSTAT3^*; *Ubc^ERT2Cre/+^* macrophages in the presence of LPS compared with those in their parental cells (**Figures 5A-C**), suggesting that after being challenged by LPS, the *Rosa26^LSL-MLS-mSTAT3^*;*Ubc^ERT2Cre/+^* macrophages did not rely on glycolysis for energy production. Similarly, in the presence of LPS, basal respiration and maximal respiration were decreased in these macrophages (**Figures 5D-E**). These observations indicate that the intrinsic expression of key factors in macrophages could influence metabolic reprogramming. Additionally, diverse microenvironments could induce the different polarization and metabolism of macrophages.

The literature indicates that in obesity, macrophages scavenge lipids and generate lipid derivatives, thereby promoting the production of inflammatory cytokines 52, 53. However, the utilization of fatty acids is also implicated in mediating inflammation. A recent study found that HDAC3 translocated to mitochondria, leading to the restriction of FAO-mediated OXPHOS 2, implying that mitochondrial OXPHOS supported by FAO has beneficial effects on immune tolerance and the suppression of innate and adaptive immunity 2, 3, 54. In contrast, our data showed that upon the LPS treatment, mitochondrial STAT3 triggered the utilization of fatty acids for the production of ATP in parallel with stabilizing CPT1a (**Figures 5-8**). These observations suggest that upon LPS treatment, mitochondrial STAT3 could induce CPT1a-USP50 binding by mediating USP50 expression, leading to CPT1a stabilization, an increase in FAO and higher ATP production. This process could, in turn, further promote NF-κB nuclear localization and inflammatory responses (**Figure 8K**).

We and others have shown that in contrast to p-STAT3 (Try705), which functions as a transcription factor mediating genes involved in proliferation and anti-apoptosis, the exposure of BMDMs to LPS induced the phosphorylation of STAT3 on Ser727 within 30 min (**Figure S2D**) and translocation into mitochondria, thereby promoting mitochondrial metabolism and mediating ROS production 31, 55. Furthermore, STAT3 was phosphorylated at the tyrosine 705 residue when the LPS stimulation was prolonged to approximately 65 min (**Figure S2D**), suggesting that p-STAT3 (Try705) could be a secondary and indirect activator.

In addition, in this study, unexpectedly, we found that mitochondrial p-STAT3 (Ser727) triggered FAO in the presence of LPS, indicating that upon TLR ligands, rapidly induced p-STAT3 (Ser727) could be a key “starter” in mitochondria, the energy factory, to ensure a rapid increase in demand for energy supply and essential metabolites.

When STAT3 is phosphorylated at the Ser727 residue, the mitochondrial complex I subunit GRIM-19 also directly interacts with STAT3 and acts as a chaperone to recruit STAT3 to mitochondria 56, 57. Silencing STAT3 in THP-1 cells or the knockdown of GRIM-19 in THP-1 cells inhibits NLRP3 inflammasome activation, indicating that mitochondrial STAT3 could be involved in the development of NLRP3-mediated inflammatory disease 58. It has been shown that high plasma concentrations of amino acids are closely related to insulin resistance. In mouse primary hepatocytes and human hepatocarcinoma HepG2 cells, excess amino acids decrease insulin-stimulated Akt phosphorylation and glycogen synthesis along with the accumulation of mitochondrial STAT3 59, 60. Silencing STAT3 rescues the insulin sensitivity inhibited by amino acids 60. Furthermore, an increase in the levels of mitochondrial STAT3 is observed in imatinib-persistent K562 cells in parallel with increased glycolysis and increased fatty acid accumulation compared to sensitive K562 cells 61. H_2_O_2_ obviously induces apoptosis in HeLa human cervical adenocarcinoma cells when these cells lack mitochondrial STAT3 62. The breast cancer cell Line 4T1 expressing STAT3^S727A^ carrying a serine-to-alanine substitution at codon 727 displays slower tumor growth and metastatic ability, lower levels of complex I activity, and increased ROS accumulation compared with their parental cells 59, 63. These observations suggest that targeting mitochondrial STAT3 has been considered a strategy to modulate biological functions 11.

Recently, several known STAT3 inhibitors have been shown to target the effects of mitochondrial STAT3. MDC-1112 reduces the accumulation of mitochondrial STAT3 and induces apoptosis in pancreatic cancer cells 64. In-house mitochondria-targeted tamoxifen (MitoTam) could suppress the phosphorylation of nuclear and mitochondrial STAT3, leading to proliferation inhibition and apoptosis in triple-negative breast cancer (TNBC) *in vitro* and *in vivo* 65. Additionally, OPB-51602 results in mitochondrial dysfunction and cell death in human prostate cancer cells by interference with mitochondrial STAT3 66. Targeting mitochondrial STAT3 by Mitocur-1 or Mitocur-3 reduces the exocytosis of mast cells and cytokine production 67. These observations suggest that targeting mitochondrial STAT3 could be a promising therapeutic strategy for cancer or allergy-associated diseases. However, the potential therapeutic value of targeting mitochondrial STAT3 with inhibitors in individuals with sepsis remains unknown.

Curcumin suppresses the nuclear STAT3 and NF-κB signaling pathways in tumors. In previous studies, curcumin has been investigated most extensively as a treatment for obesity and obesity-related metabolic diseases 68. We sought to examine whether curcumin could inhibit mitochondrial STAT3 activity *in vitro* and *in vivo*. As expected, curcumin attenuated the phosphorylation of STAT3 (Ser727) mediated by either LPS or INF-α (**Figure 8G**). Curcumin also decreased the levels of p-STAT3 (Tyr705) induced by INF-α (**Figure 8G**). Moreover, curcumin reduced LPS-enhanced FAO in mitochondrial STAT3 knock-in macrophages (**Figure 8I**). Importantly, curcumin extended the survival curve of the LPS-induced sepsis mouse model (**Figures 8A and 8F**). Our study results further suggest that the pharmacological blockade of mitochondrial STAT3 function by curcumin could be a promising treatment strategy for sepsis, metabolic disorders, and other inflammatory diseases.

## Limitations and Future Directions

Although our study demonstrates that in the presence of LPS, mitochondrial STAT3 drives FAO by inducing CPT1a stabilization mediated by USP50 in macrophages, several questions emerge from our study. Regarding nuclear NF-κB expression in the presence of ATP or LPS (**Figures 7I and 7J**), it seems that the effect of ATP and LPS on NF-κB nuclear expression is stronger than that of mitochondrial STAT3 alone *in vitro*, leading us to consider whether sepsis or metabolic disorder patients can benefit from mitochondrial STAT3 inhibitors, although we cannot exclude the possibility that the difference between ATP/LPS-induced NF-κB expression and mitochondrial STAT3-mediated nuclear NF-κB expression is due to differences in the treatment conditions. Unfortunately, we could not test this hypothesis because of the unavailability of specific mitochondrial STAT3 inhibitors. In addition, are other factors involved in mediating USP50 expression in LPS-treated mitochondrial STAT3 macrophages? Because the role of FAO in macrophage activation and polarization is still under scrutiny 2, mitochondrial STAT3-triggered FAO is involved in inflammasome activation. The effects of curcumin in sepsis patients need to be investigated in future studies. Since neutrophils also play an important role in sepsis, does mitochondrial STAT3 affect neutrophil function in the development of sepsis?

## Supplementary Material

Supplementary figures.

## Figures and Tables

**Table 1:** Real-time RT-PCR primers.

Gene	Direction	Primer
*Il1b*	Forward	5'-CAGGCAGGCAGTATCACTCA-3'
	Reverse	5'-TGTCCTCATCCTGGAAGGTC-3'
* Il6*	Forward	5'-CCGGAGAGGAGACTTCACAG-3'
	Reverse	5'-TCCACGATTTCCCAGAGAAC-3'
*Cpt1a*	Forward	5'-GAACCCCAACATCCCCAAAC-3'
	Reverse	5'-AACTGGCACTGCTTAGGGAT-3'
*Actb*	Forward	5'-GCTACAGCTTCACCACCACA-3'
	Reverse	5'-TCTCCAGGGAGGAAGAGGAT-3'
*Irf8*	Forward	5'-CAATCAGGAGGTGGATGCTT-3'
	Reverse	5'-GGCTGGTTCAGCTTTGTCTC-3'
*Ms4a3*	Forward	5'-GAAGCCAGAGGAGACTGGTG-3'
	Reverse	5'-GAGGGCTTGCAGTACACCTC-3'
*Plbd1*	Forward	5'-ATCCCTGCAGTACCATCTGC-3'
	Reverse	5'-AAAGATGTCTGCCACCTTGG-3'
*Lgals3*	Forward	5'-GATCACAATCATGGGCACAG-3'
	Reverse	5'-GTGGAAGGCAACATCATTCC-3'
*Mtpn*	Forward	5'-ACGGAGACTTGGATGAGGTG-3'
	Reverse	5'-TCAAGCTGTCCACAATCTGC-3'
*Atp5a1*	Forward	5'-GCCCTCGGTAATGCTATTGA-3'
	Reverse	5'-CACAGAGATTCGGGGGATAA-3'
*Atp5g3*	Forward	5'-CGAAGGGAGTTTCAGACCAG-3'
	Reverse	5'-CACCAGAACCAGCAACTCCT-3'
*Tsc22d3*	Forward	5'-GGTGGCCCTAGACAACAAGA-3'
	Reverse	5'-TCTTCTCAAGCAGCTCACGA-3'
*Hsp90aa1*	Forward	5'-AAGGCAGAGGCTGACAAGAA-3'
	Reverse	5'-CTGGGGATCTTCCAGACTGA-3'
*Nfkbia*	Forward	5'-TGGCCAGTGTAGCAGTCTTG-3'
	Reverse	5'-GACACGTGTGGCCATTGTAG-3'
*Nfkbiz*	Forward	5'-CAGTTGCCTGTCTTTCGTGA-3'
	Reverse	5'-TCCAACTGTGTCACCCGATA-3'
*Usp50*	Forward	5'-TAAGACCCAGCCACACTTCC-3'
	Reverse	5'-AGATACGCTGCAGAGGCATT-3'
*mNd1*	Forward	5'-CCATTCTAATCGCCATAGCC-3'
	Reverse	5'-ATGCCGTATGGACCAACAAT-3'
*mPuma*	Forward	5'-CAAGAAGAGCAGCATCGACA-3'
	Reverse	5'-TAGTTGGGCTCCATTTCTGG-3'

**Table 2:** The protein in mitochondria fraction.

Gene	Protein name	Peptides	Coverage
LDHA	L-lactate dehydrogenase A	12	58.4
HSPD1	Heat shock 60kDa protein 1	5	30.5
ETFA	Electron transfer flavoprotein subunit alpha	4	42.8
STAT3	Signal transducer and activator of transcription 3	3	24.6
CYB5R3	NADH-cytochrome b5 reductase 3	1	28.6
