# Supplementary material for: Mitochondrial STAT3 exacerbates LPS-induced sepsis by driving CPT1a-mediated fatty acid oxidation
Source: Theranostics. 2022 Jan 1;12(2):976–98. doi: 10.7150/thno.63751 (PMC8692896; doi:10.7150/thno.63751)
Supplement: Supplementary file 1 — Supplementary figures. [file thnov12p0976s1.pdf]

Supplementary Figure Legends

Figure S1. The levels of STAT3 in mitochondria of phagocytes or tissues from inducible mitochondrial STAT3 knock-in mice, as shown in Figure. 1

(A) The amounts of STAT3 were quantified by densitometry, normalized to HSP60, and plotted. **(B)** The relative amounts of STAT3 in the indicated cell fractionation were quantified, normalized to the indicated loading marker, and plotted. (A and B) The data shown represent a combination of three independent experiments. ** *P* < 0.01. **(C)** Immunofluorescence of p-STAT3 (Ser727, gray), p-STAT3 (Tyr705, red), HSP60 (green), and DAPI (blue) in MEFs. The data are representative of one of three independent experiments. **(D)** The relative amounts of STAT3 in the livers, intestines or colons were quantified, normalized to actin, and compared. The data shown represent a combination of three independent experiments and were compared using Multiple t test–one per row. ** *P* < 0.01. **(E)** The STAT3 expression was assessed in livers and colons harvested from the indicated mice by a Western blot analysis. The relative amounts of STAT3 in these tissues were quantified and normalized to actin, and plotted.

Figure S2. Mitochondrial STAT3 didn't influence the development of HSC, as shown in Figures. 1 and 2

(A) 6-week-old mice were treated with 4-OHT for 7 times. 2 days later, the development of HSC with different genotypes was assessed by flow cytometry. The data shown were representative of one of two independent experiments.

(B) The graph shows the percentage of indicated cells in BM gating strategy in (A). The data shown was representative of one of two independent experiments (n = 7 mice/group) with similar results. Significance was calculated using an unpaired Student's t-test. **(C)** The graph shows the number of indicated cells in BM gating strategy in (A). The data shown was representative of one of two independent experiments (n = 7 mice/group) with similar results. Significance was calculated using an unpaired Student's t-test. **(D)** BMDMs were stimulated with or without 100 ng/mL LPS for the indicated time point. p-STAT3 (Ser727),

p-STAT3 (Tyr705), STAT3 and actin were analyzed by a Western blot analysis. The relative levels of the indicated protein were quantified, normalized to actin, and plotted. The plot data shown represent a combination of three independent experiments and were compared using two-way ANOVA followed by multiple comparisons. ** $P < 0.01$, * $P < 0.05$.

Figure S3. The genes involved in mediating inflammation and metabolism were validated by real-time RT–PCR, as shown in Figure. 3

(A and B) RNA was extracted and cDNA was synthesized, and subjected to real-time RT–PCR. Data are shown as the mean ± SD and are representative of one of two independent experiments performed in triplicate. ** $P < 0.01$, * $P < 0.05$. **(C)** Induced BMDMs were treated with 100 nM 4-OHT for 3 days. Nuclear, cytoplasmic, and mitochondrial fractions were extracted. The amounts of indicated protein were quantified by densitometry, normalized to loading control, and plotted. The data shown represent a combination of three independent experiments. ** $P < 0.01$.

Figure S4. Mitochondrial STAT3 was involved in mediating CPT1a stabilization, as shown in Figure. 6

(A-B) The amounts of indicated protein were quantified by densitometry, normalized to loading control, and plotted. The data shown represent a combination of three independent experiments. ** $P < 0.01$.

Figure S5. USP50 is critical for CPT1a stabilization, as shown in Figure. 7

(A) BMDMs of the indicated genotypes were treated with 4-OHT for 3 days, followed by treatment with 100 ng/mL LPS for an additional 6 h before harvesting. The levels of indicated protein were examined. **(B)** $Rosa26^{LSL\text{-}MLS\text{-}Stat3}$ macrophages were transfected with a negative control siRNA or a siRNA against USP50. The levels of CPT1a, USP50 and actin were assessed. **(C)** The levels of p65 in the indicated cell fractionation were examined. (A-C) The amounts of indicated protein were quantified by densitometry, normalized to loading control, and plotted. The data shown represent a combination of three

independent experiments. ** *P* < 0.01, n.s., not significant.

Figure S6. Curcumin suppressed the activation of STAT3 and NF-κB, as shown in Figure. 8

The levels of serum LDH **(A)** and albumin **(B)** were assessed. (A and B) The data shown are representative of one of two independent experiments. Numerical data are shown as the mean ± SD. ** *P* < 0.01, * *P* < 0.05. **(C-F)** The amounts of indicated protein were quantified by densitometry, normalized to loading control, and plotted. The data shown represent a combination of three independent experiments. ** *P* < 0.01, * *P* < 0.05.

Figure S1

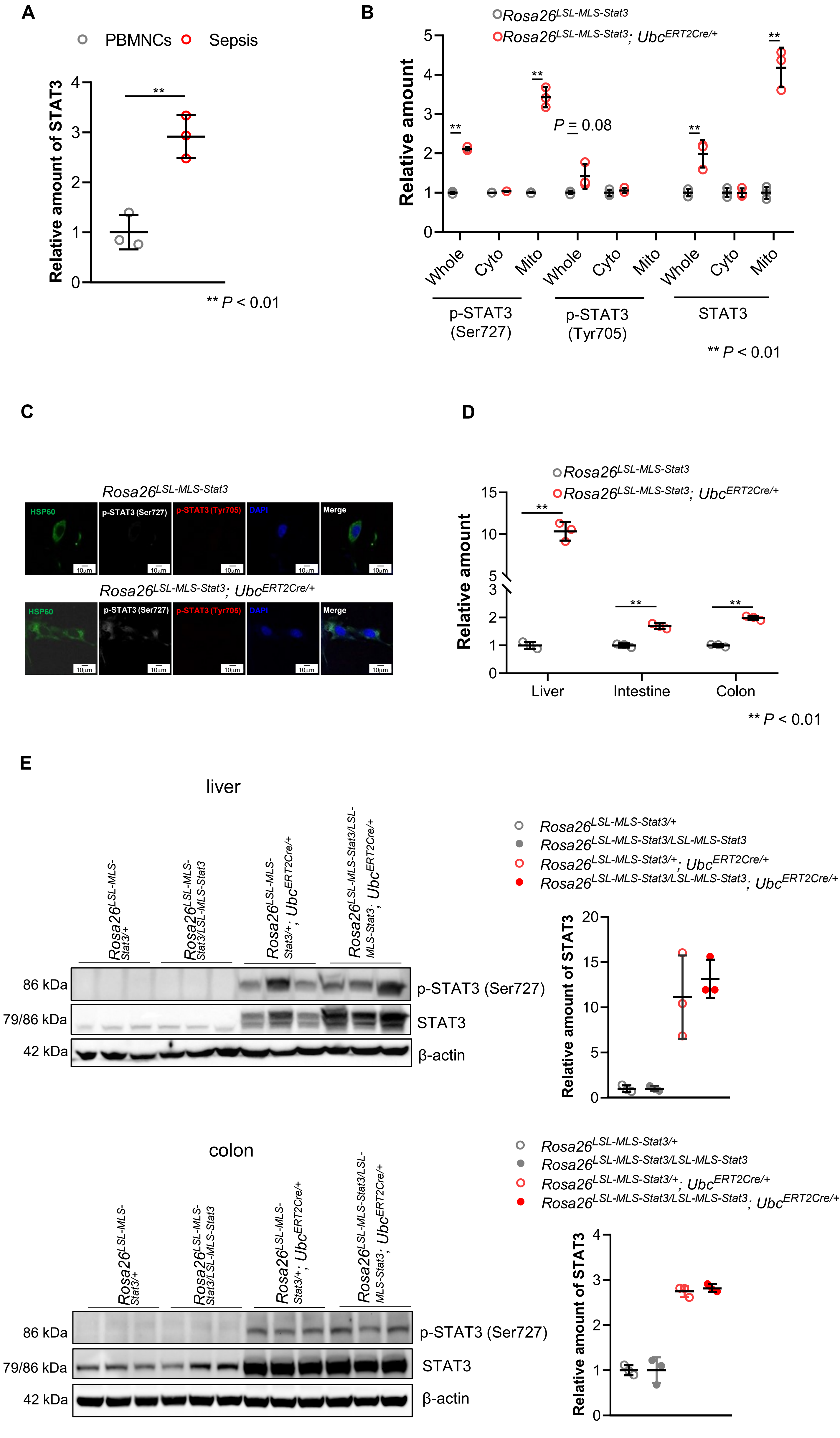
A
PBMNCs
Sepsis
Relative amount of STAT3
** P < 0.01
B
Rosa26LSL-MLS-Stat3
Rosa26LSL-MLS-Stat3; UbcERT2Cre/+
Relative amount
P = 0.08
Whole
Cyto
Mito
p-STAT3 (Ser727)
p-STAT3 (Tyr705)
STAT3
** P < 0.01
C
Rosa26LSL-MLS-Stat3
Rosa26LSL-MLS-Stat3; UbcERT2Cre/+
HSP60
p-STAT3 (Ser727)
p-STAT3 (Tyr705)
DAPI
Merge
10μm
D
Rosa26LSL-MLS-Stat3
Rosa26LSL-MLS-Stat3; UbcERT2Cre/+
Relative amount
Liver
Intestine
Colon
** P < 0.01
E
liver
Rosa26LSL-MLS-Stat3/+
Rosa26LSL-MLS-Stat3/LSL-MLS-Stat3
Rosa26LSL-MLS-Stat3/+; UbcERT2Cre/+
Rosa26LSL-MLS-Stat3/LSL-MLS-Stat3; UbcERT2Cre/+
86 kDa
79/86 kDa
42 kDa
p-STAT3 (Ser727)
STAT3
β-actin
Relative amount of STAT3
colon

Figure S2

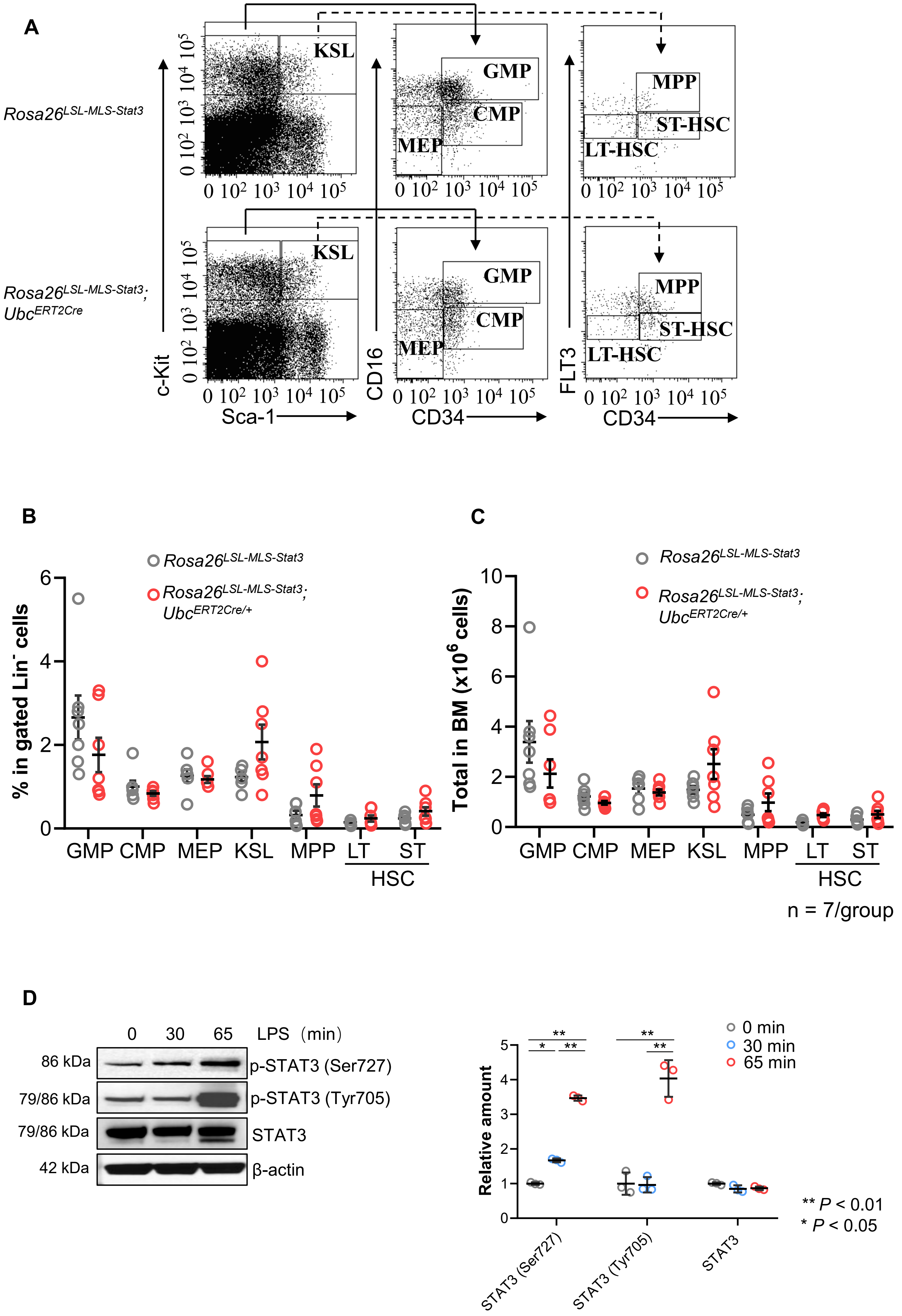
A
Rosa26LSL-MLS-Stat3
Rosa26LSL-MLS-Stat3; UbcERT2Cre
KSL
GMP
CMP
MEP
MPP
ST-HSC
LT-HSC
c-Kit
Sca-1
CD16
CD34
FLT3
B
Rosa26LSL-MLS-Stat3
Rosa26LSL-MLS-Stat3; UbcERT2Cre/+
% in gated Lin- cells
GMP CMP MEP KSL MPP LT ST
HSC
C
Rosa26LSL-MLS-Stat3
Rosa26LSL-MLS-Stat3; UbcERT2Cre/+
Total in BM (x10^6 cells)
GMP CMP MEP KSL MPP LT ST
HSC
n = 7/group
D
0 30 65 LPS (min)
86 kDa p-STAT3 (Ser727)
79/86 kDa p-STAT3 (Tyr705)
79/86 kDa STAT3
42 kDa β-actin
Relative amount
0 min
30 min
65 min
STAT3 (Ser727)
STAT3 (Tyr705)
STAT3
** P < 0.01
* P < 0.05

Figure S3

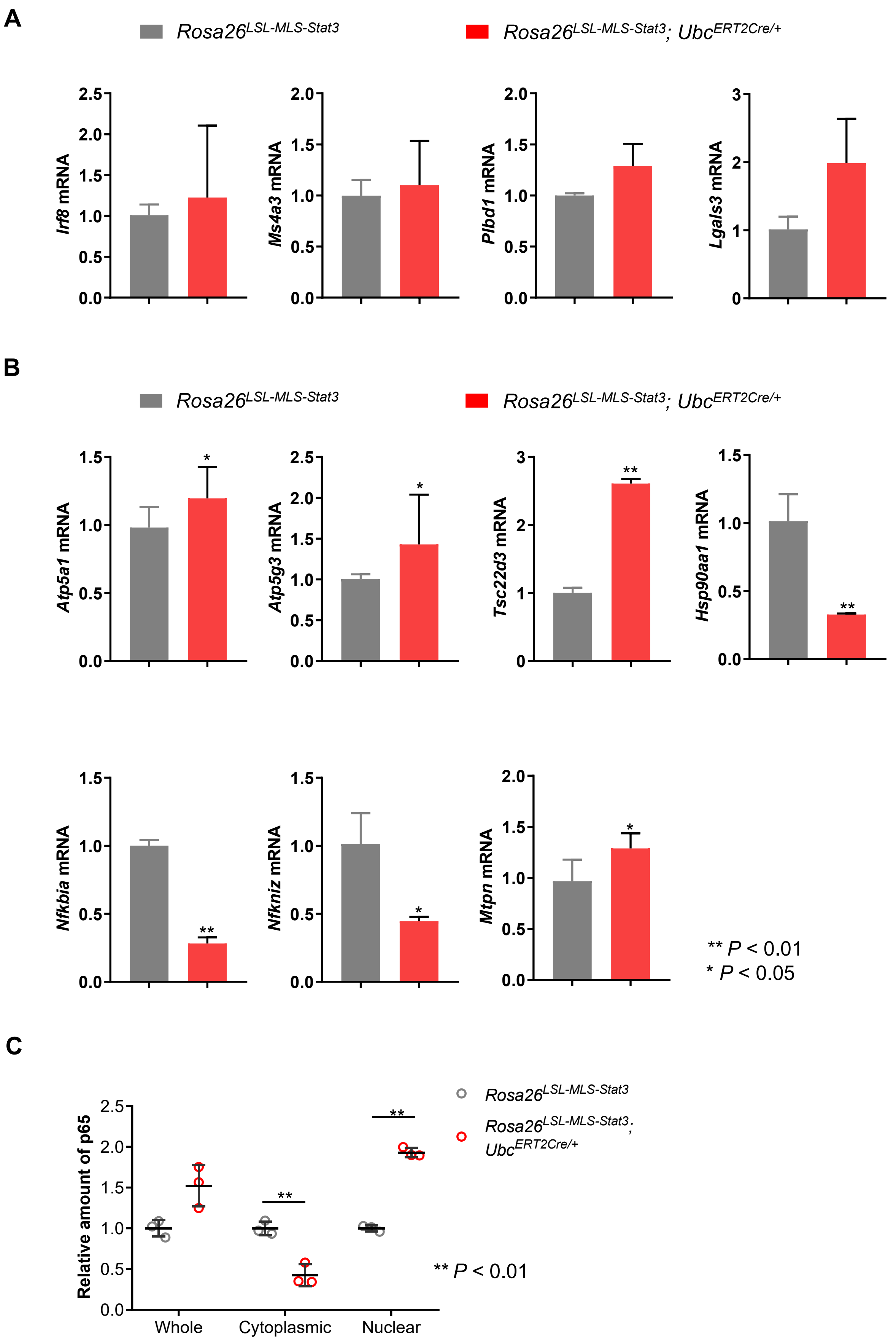
A
Rosa26LSL-MLS-Stat3
Rosa26LSL-MLS-Stat3; UbcERT2Cre/+
Irf8 mRNA
Ms4a3 mRNA
Plbd1 mRNA
Lgals3 mRNA
B
Rosa26LSL-MLS-Stat3
Rosa26LSL-MLS-Stat3; UbcERT2Cre/+
Atp5a1 mRNA
Atp5g3 mRNA
Tsc22d3 mRNA
Hsp90aa1 mRNA
Nfkbia mRNA
Nfkniz mRNA
Mtpn mRNA
** P < 0.01
* P < 0.05
C
Relative amount of p65
Whole
Cytoplasmic
Nuclear
Rosa26LSL-MLS-Stat3
Rosa26LSL-MLS-Stat3; UbcERT2Cre/+
** P < 0.01

Figure S4

A

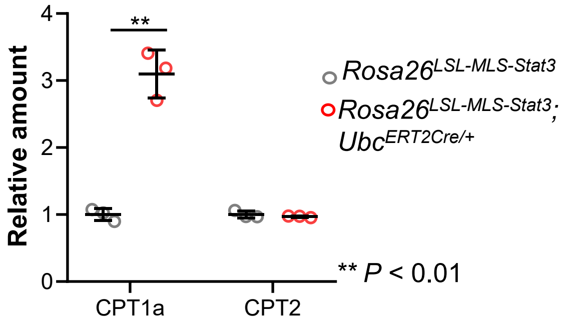
Relative amount
4
3
2
1
0
**
CPT1a
CPT2
Rosa26LSL-MLS-Stat3
Rosa26LSL-MLS-Stat3;
UbcERT2Cre/+
** P < 0.01

B

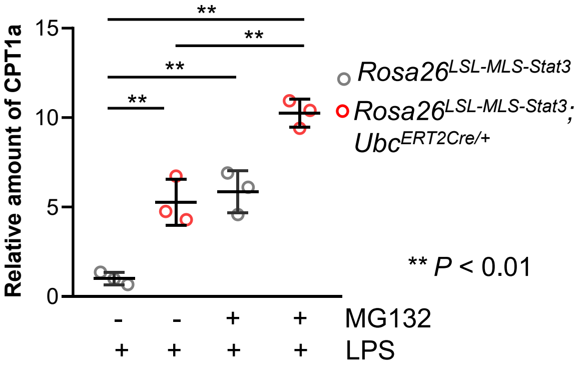
Relative amount of CPT1a
15
10
5
0
**
**
**
**
Rosa26LSL-MLS-Stat3
Rosa26LSL-MLS-Stat3;
UbcERT2Cre/+
** P < 0.01
- - + + MG132
+ + + + LPS

Figure S5

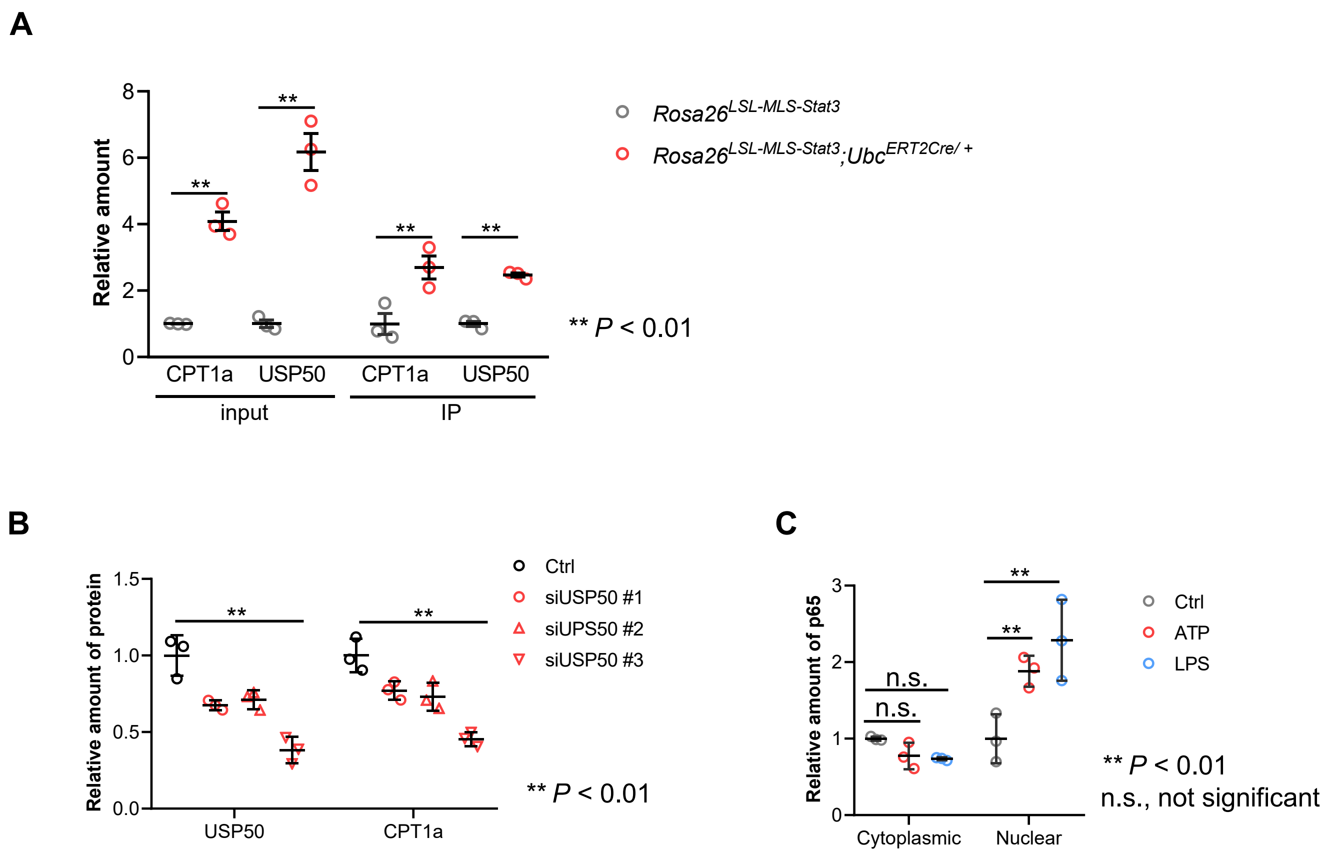
A
Relative amount
8
6
4
2
0
**
**
**
**
CPT1a
USP50
CPT1a
USP50
input
IP
Rosa26LSL-MLS-Stat3
Rosa26LSL-MLS-Stat3;UbcERT2Cre/+
** P < 0.01
B
Relative amount of protein
1.5
1.0
0.5
0.0
**
**
USP50
CPT1a
Ctrl
siUSP50 #1
siUPS50 #2
siUSP50 #3
** P < 0.01
C
Relative amount of p65
3
2
1
0
n.s.
n.s.
**
**
Cytoplasmic
Nuclear
Ctrl
ATP
LPS
** P < 0.01
n.s., not significant

Figure S6

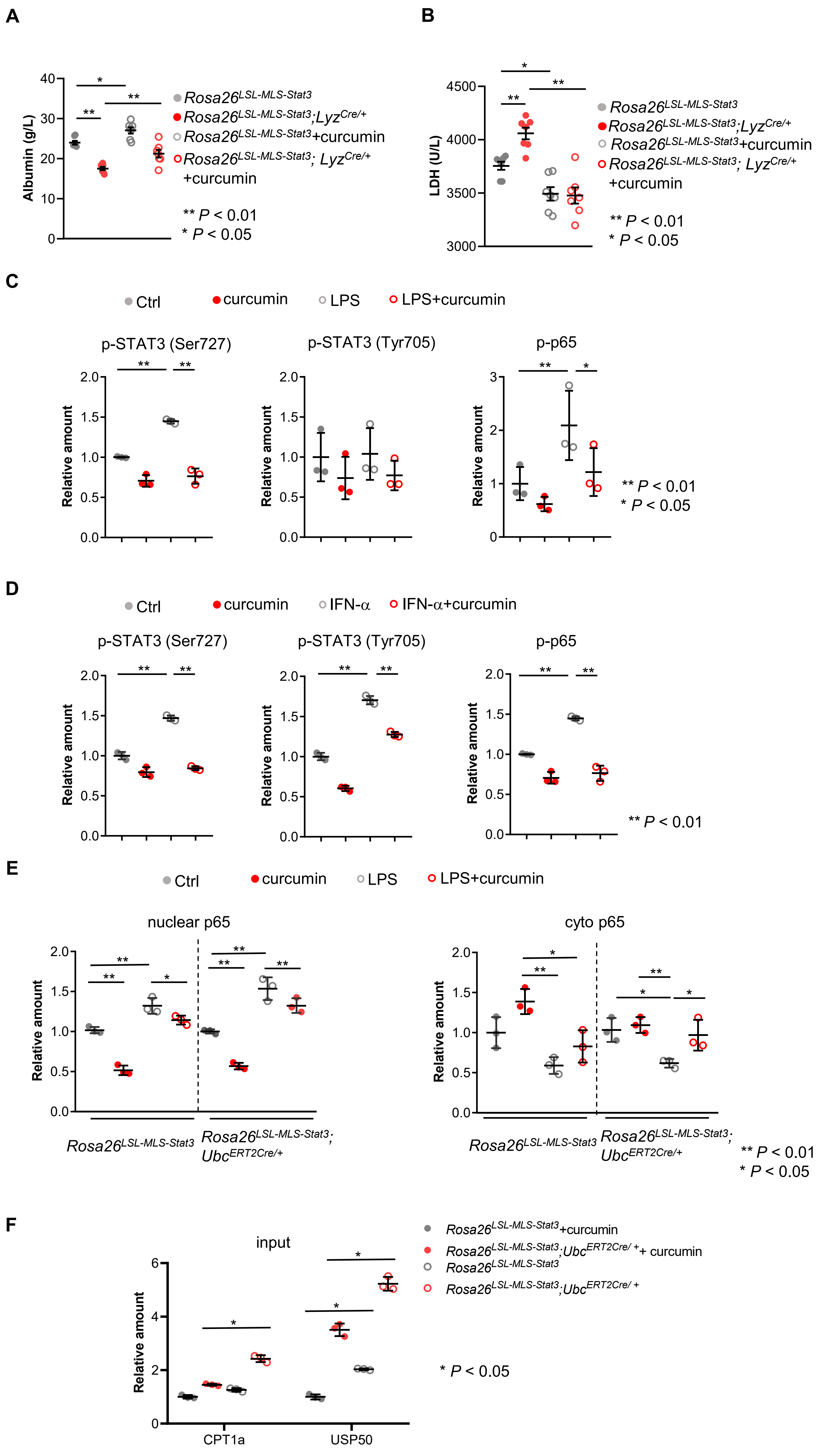
A
Albumin (g/L)
Rosa26LSL-MLS-Stat3
Rosa26LSL-MLS-Stat3;LyzCre/+
Rosa26LSL-MLS-Stat3+curcumin
Rosa26LSL-MLS-Stat3; LyzCre/+ +curcumin
** P < 0.01
* P < 0.05
B
LDH (U/L)
Rosa26LSL-MLS-Stat3
Rosa26LSL-MLS-Stat3;LyzCre/+
Rosa26LSL-MLS-Stat3+curcumin
Rosa26LSL-MLS-Stat3; LyzCre/+ +curcumin
** P < 0.01
* P < 0.05
C
Ctrl
curcumin
LPS
LPS+curcumin
p-STAT3 (Ser727)
p-STAT3 (Tyr705)
p-p65
Relative amount
** P < 0.01
* P < 0.05
D
Ctrl
curcumin
IFN-α
IFN-α+curcumin
p-STAT3 (Ser727)
p-STAT3 (Tyr705)
p-p65
Relative amount
** P < 0.01
E
Ctrl
curcumin
LPS
LPS+curcumin
nuclear p65
cyto p65
Relative amount
Rosa26LSL-MLS-Stat3
Rosa26LSL-MLS-Stat3; UbcERT2Cre/+
** P < 0.01
* P < 0.05
F
input
Relative amount
CPT1a
USP50
Rosa26LSL-MLS-Stat3+curcumin
Rosa26LSL-MLS-Stat3;UbcERT2Cre/+ + curcumin
Rosa26LSL-MLS-Stat3
Rosa26LSL-MLS-Stat3;UbcERT2Cre/+
* P < 0.05
